# Seventy Years of *Chlamydia* Vaccine Research – Limitations of the Past and Directions for the Future

**DOI:** 10.3389/fmicb.2019.00070

**Published:** 2019-01-31

**Authors:** Samuel Phillips, Bonnie L. Quigley, Peter Timms

**Affiliations:** Genecology Research Centre, The University of the Sunshine Coast, Maroochydore, QLD, Australia

**Keywords:** *Chlamydia*, vaccine, MOMP (major outer membrane protein), mice, koala (*Phascolarctos cinereus*)

## Abstract

*Chlamydia* is a major bacterial pathogen that infects humans, as well as a wide range of animals, including marsupials, birds, cats, pigs, cattle, and sheep. Antibiotics are the only treatment currently available, however, with high rates of re-infection, there is mounting pressure to develop *Chlamydia* vaccines. In this review, we analyzed how *Chlamydia* vaccine trials have developed over the past 70 years and identified where future trials need to be focused. There has been a strong bias toward studies targeting *C. muridarum* and *C. trachomatis* within mice and a lack of studies matching chlamydial species to their end target host. Even though a large number of specific antigenic targets have been studied, the results from whole-cell vaccine targets show slightly more promising results overall. There has also been a strong bias toward systemic vaccine delivery systems, despite the finding that mucosal delivery systems have shown more promising outcomes. However, the only successful vaccines with matched chlamydial species/infecting host are based on systemic vaccine delivery methods. We highlight the extensive work done with mouse model trials and indicate that whole cell antigenic targets are capable of inducing an effective response, protecting from disease and reducing shedding rates. However, replication of these results using antigen preparations more conducive to commercial vaccine production has proven difficult. To date, the Major Outer Membrane Protein (MOMP) has emerged as the most suitable substitute for whole cell targets and its delivery as a combined systemic and mucosal vaccine is most effective. Finally, although mouse model trials are useful, differences between hosts and infecting chlamydial strains are preventing vaccine formulations from mouse models to be translated into larger animals or intended hosts.

## Introduction

**FIGURE 1:**
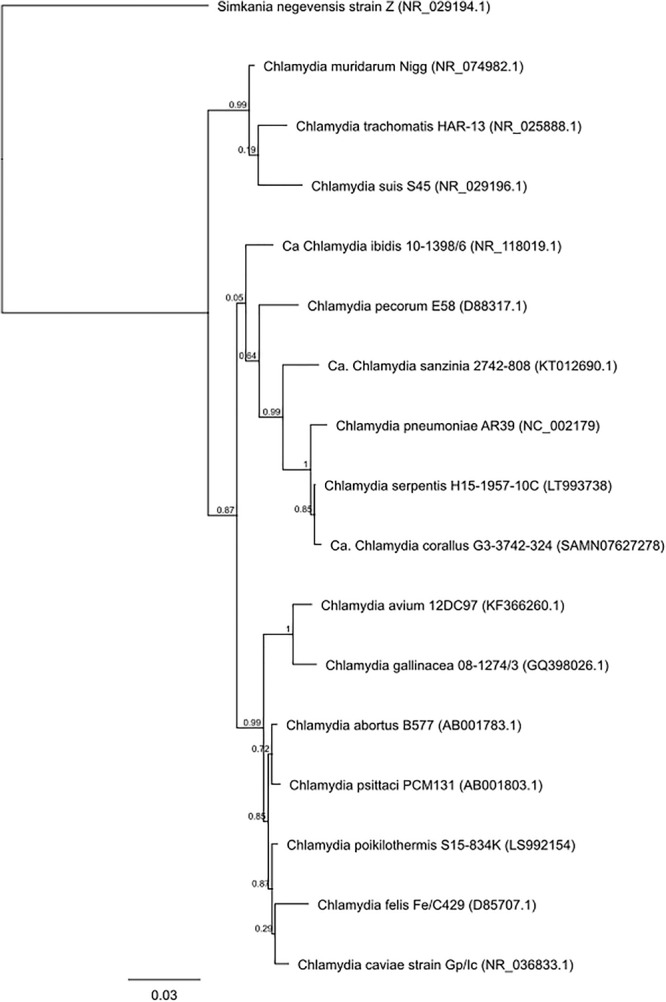
Phylogenetic tree analysis of the family Chlamydiaceae. Approximate likelihood phylogenetic tree analysis, MAFFT alignment of NCBI (Genbank) 16S sequences (1,587 bp) assembled using Geneious v 11.1.4.

Chlamydiae are gram-negative, obligate intracellular pathogens that infect eukaryotic cells (Oehme et al., 1991). There are currently 16 classified and / or formally proposed species that comprise the *Chlamydiaceae* family and these species infect a wide range of hosts and anatomical sites (Table 1 and Figure 1) (Taylor-Brown and Polkinghorne, 2017). Vaccines are being developed to target some of these chlamydial species for a variety of reasons (Table 2). Vaccines targeting human pathogens are designed to protect human health, while vaccines targeting livestock and wildlife pathogens aim to prevent economic damage, protect endangered animals and prevent zoonotic disease transmission. Although these 16 species of *Chlamydia* infect a range of different hosts, the site of infection and disease pathology within hosts are highly similar, indicating commonalities between a seemingly diverse group of chlamydial organisms.

**Table 1:** Species and known hosts of the *Chlamydiaceae* family

Species	Predominant host	Site of disease
*C. trachomatis*	Human	Urogenital and Conjunctiva
*C. pneumoniae*		Respiratory
*C. caviae*	Guinea pig	Urogenital, Conjunctiva and Respiratory
*C. muridarum*	Mice	Urogenital
*C. psittaci*	Bird	Respiratory and Placenta
*C. avium*		Respiratory
*C. ibidis*		
*C. gallinacea*		
*C. suis*	Pig	Urogenital and Conjunctiva
*C. felis*	Cat	Urogenital, Conjunctiva and Respiratory
*C. abortus*	Livestock^∗^	Placenta
*C. pecorum*	Marsupials and livestock^∗^	Urogenital and Conjunctiva
*C. serpentis*	Snake	Cloacal and choanal
*C. poikilothermis*		
*C. corallus*		
*C. sanzinia*	Snake and turtle	Cloacal and Respiratory

*^∗^Sheep, cattle, goat, and horse.*

**Table 2:** Targeted host and the number of trials for each, separated by chlamydial strain.

Host	*Chlamydia* strains (number of studies)	Purpose of trials	Total number of trials
Mice/Rats	*C. muridarum* (82),	*Human vaccine targeting C. trachomatis or C. pneumoniae*	160
	*C. trachomatis* (60),		
	*C. pneumoniae* (14),		
	*C. psittaci* (8),		
	*C. abortus* (6),	Sheep vaccine targeting *C. abortus*	
	*C. pecorum* (1)	Sheep vaccine targeting *C. pecorum*	
Non-Human primates	*C. muridarum* (2), *C. trachomatis* (10)	Human vaccine targeting *C. trachomatis*	11
Guinea pigs	*C. psittaci* (4), *C. trachomatis* (3), *C. caviae* (1)	Human vaccine targeting *C. trachomatis* or *C. pneumoniae*	6
Humans	*C. trachomatis* (1)	Human vaccine targeting *C. trachomatis*	1
Rabbits	*C. trachomatis* (1)	Human vaccine targeting *C. trachomatis*	1
Pigs	*C. abortus* (2)	Pig vaccine targeting *C. abortus*	7
	*C. trachomatis (5)*	Human vaccine targeting *C. trachomatis*	
Cattle	*C. abortus* (1)	Cattle vaccine targeting *C. abortus*	1
Sheep	*C. abortus* (3), *C. pecorum* (1), *C. psittaci* (9)	Sheep vaccine targeting *C. abortus, C. pecorum*, or *C. psittaci*	13
Birds	*C. psittaci* (5)	Bird vaccine targeting *C. psittaci*	5
Cats	*C. felis* (1), *C. psittaci* (1)	Cat vaccine targeting *C. felis*	2
Koalas	*C. pecorum* (11)	Koala vaccine targeting *C. pecorum*	11

### Human Pathogenic Species

*Chlamydia trachomatis* has been dived into 13 different genotypes based on the major outer membrane protein (MOMP) (Stevens et al., 2010). Genotypes A, B, and C infect the conjunctiva of humans leading to active and scarring trachoma and eventually blindness (Garland et al., 1995). Genotypes D – K and L1 – L3 predominantly infect the urogenital tract, leading to inflammation, scarring and infertility. In women, these genotypes can also result in pelvic inflammatory disease, which increases the risk of ectopic pregnancy (Menon et al., 2015). It has been reported that up to 80% of *C. trachomatis* infections are asymptomatic (no signs of pathology), resulting in individuals who are unaware they are infected and leading to an extremely high rate of transmission (Korenromp et al., 2002; Farley et al., 2003; Ljubin-Sternak and Mestrovic, 2014; Menon et al., 2015).

*Chlamydia pneumoniae* predominantly infects the respiratory tract of humans leading to pneumonia (Shi et al., 2002; Kurz et al., 2009) as well as having some links to atherosclerosis, Alzheimer’s disease and asthma (Balin et al., 1998; Daba et al., 2002; Deniset et al., 2010; Iramain et al., 2016). In addition, *C. pneumoniae* has been reported in a range of animals such as mice, pigs, marsupials, birds, cats, and livestock, leading to respiratory disease (Borel et al., 2018).

### Animal Pathogenic Species With Zoonotic Potential

Other species of *Chlamydia* infect a wide range of animals leading to disease and reported zoonotic potential (Li et al., 2017; Jelocnik et al., 2018; Pisanu et al., 2018; Torres-Mejía et al., 2018).

*Chlamydia psittaci* is a respiratory and reproductive pathogen of birds with zoonotic potential for humans. *C. psittaci* disease (psittacosis) outbreaks in humans date back to 1879 where humans were infected from pet parrots and finches. In the 1930s, human pandemic outbreaks were linked to racing pigeons imported from South America to Europe and North America. More recently, human psittacosis outbreaks throughout Europe have been linked to turkey and duck farming (Beeckman and Vanrompay, 2009). Broadly, a recent review and meta-analysis demonstrated that *C. psittaci* is the causative agent in 1% of worldwide community-acquired pneumonia (Hogerwerf et al., 2017).

*Chlamydophila abortus* predominantly infects the placenta of livestock resulting in fetal death and has the zoonotic potential to cause abortions in women if infected during pregnancy (Szeredi and Bacsadi, 2002; DeGraves et al., 2004; Meijer et al., 2004; Masala et al., 2007).

*Chlamydophila felis* infects the respiratory tract and conjunctiva of cats, leading to respiratory disease and conjunctivitis, respectively (Sykes, 2001; Cai et al., 2002; Rampazzo et al., 2003). *C. felis* has also been reported in as many as eight different zoonotic transmission events, however, these all occurred within immunocompromised humans (Browning, 2004). Other chlamydial species identified with zoonotic potential include *C. caviae* in guinea pigs, where three unrelated, zoonotic transmission events were reported in healthy adult humans, presenting with respiratory failure due to severe community-acquired pneumonia (Ramakers et al., 2017). Finally, *C. suis* has been detected from farm workers who have close contact to pigs, however no clinical symptoms of illness has yet been reported, so further research is needed to evaluate the potential risk to people (De Puysseleyr et al., 2017).

### Other Animal Pathogenic Species

*Chlamydia pecorum* is one of the most diverse chlamydial species and can be separated into two genetically distinct clades based on host species. The first clade of *C. pecorum* infects the conjunctiva, limb joints and urogenital tract of livestock, leading to conjunctivitis, arthritis, cystitis and the development of reproductive cysts resulting in infertility (Reinhold et al., 2011; Jelocnik et al., 2014; Walker et al., 2016, 2018; Bommana et al., 2018). Furthermore, gastrointestinal infections have been shown to cause overall health deterioration, including increased body temperature and decreased body weights (Reinhold et al., 2008, 2011; Li et al., 2016). The second clade of *C. pecorum* infect the conjunctiva, and urogenital tract of predominantly koalas, leading to keratoconjunctivitis, cystitis and the development of reproductive cysts resulting in infertility (Burnard et al., 2017).

*Chlamydia suis* strains infect the conjunctiva, gastrointestinal tract and respiratory tract of pigs, resulting in a range of diseases including conjunctivitis (pink eye), intestinal lesions and respiratory disease (Rogers and Andersen, 2000; Sachse et al., 2004; Reinhold et al., 2005; Becker et al., 2007). *C. suis* has also been reported from pig oviducts and uteri, however links to disease development at these anatomical sites is yet to be established (Kauffold et al., 2006).

*Chlamydia avium, C. ibidis and C. gallinacea* are all predominantly infections of birds, infecting the respiratory tract and resulting in respiratory disease (Mitura et al., 2014; Guo et al., 2016; Chu et al., 2017).

*Chlamydia serpentis, C. poikilothermis, C. corallus*, and *C. sanzinia* are recently discovered species of *Chlamydia* which have been identified from cloacal and choanal samples of captive and wild snakes, with *C. sanzinia* also recently identified from cloacal and pharyngeal samples of turtles and tortoises. These newly identified species have very little known about their pathogenic potential (Taylor-Brown et al., 2016, 2017; Mitura et al., 2017; Staub et al., 2018).

### Surrogate Models

Finally, *C. muridarum* in mice and *C. caviae* in guinea pigs have been used as models for chlamydial research. These two strains infect the urogenital tract and conjunctiva of their respective hosts, leading to hydrosalpinx and conjunctivitis, respectively (Andrew et al., 2011; Wali et al., 2014; De La Maza et al., 2017).

Mice and guinea pigs are used as surrogate models as their disease pathology mirrors diseases seen in humans, they have very similar biological process to humans and can be utilized in challenge trials, testing the effect of a vaccine candidate.

### Immunological Response to Chlamydial Infections

For the successful clearance of chlamydial infections, cell-mediated and humoral immune response coordination is required. The humoral immune response to intracellular bacteria is a relatively new concept, with the common belief before 2004 being that humoral (antibody) immunity protected against extracellular bacteria and cellular immunity protected against intracellular pathogens (Casadevall, 2003, 2004). However, the use of B cell deficient mice and the identification of monoclonal antibodies have shed new light on the remarkable complexity of antibody mediated immunity (AMI) (Casadevall and Pirofski, 2006). Many studies have now shown that the appearance of serum antibodies strongly correlates with chlamydial clearance (Casadevall and Pirofski, 2006). Furthermore, the presence of IgA within human vaginal secretions correlates with chlamydial clearance (Brunham et al., 1983). However, it is still well-established that a combined humoral and cellular mediated immune response is required for complete protection from chlamydial infection and disease progression (Williams et al., 1987; Ramsey et al., 1988). The cellular immune response requires the recruitment of macrophages, dendritic cells, natural killer cells and CD4/CD8 T cells to the mucosal site of infection (Brunham and Rey-Ladino, 2005; Vasilevsky et al., 2014). The primary cytokine involved in chlamydial clearance is interferon gamma (IFNγ). Stimulated through the interleukin (IL) 12 cytokine pathway (Trinchieri, 2003; Eyerich et al., 2010), in humans, IFNγ restricts the growth cycle of *Chlamydia* by depleting tryptophan through the indoleamine 2,3-dioxygenase (IDO) pathway. IFNγ also suppresses inflammation at the site of infection through the downregulation of the Th2 immune response, characterized by IL4 and IL10 (Natividad et al., 2005). Failure to downregulate Th2 results in negative feedback, depleting IFNγ (Holland et al., 1996). IL12 also stimulates cytokines TNFα and IL22 involved in epithelial protective immunity (Trinchieri, 2003; Eyerich et al., 2010; Zhao et al., 2015). In addition to the stimulatory effects on inflammation by IL12, evidence indicates that regulatory T cells (Tregs) balance this response through counter-inflammatory pathways (Faal et al., 2006).

Clearly, there is an incredibly complex interaction between chlamydia and the host immune system. It is not possible to monitor all aspects of the immune response during each vaccine trial, so the reported protection achieved from each trial must be summarized and inferred from the data available. As such, for the purposes of this review, trials demonstrating reduced disease pathology when challenged and achieving a decrease in chlamydial shedding rates (compared to controls) were denoted as having achieved partial protection, regardless of the immune response measured (humoral or cellular). Any study reporting no disease pathology and no detectable chlamydial organisms after challenge infection was denoted as having achieved full protection. Finally, trials failing to control the onset of disease pathology were denoted as no protection.

The purpose of this review is to highlight and summarize the breadth of chlamydial based vaccine trials performed to date and, based on this summary, recommend where future vaccine trial effort should be focused.

## Overview of *Chlamydia* Vaccine Trials

**FIGURE 2:**
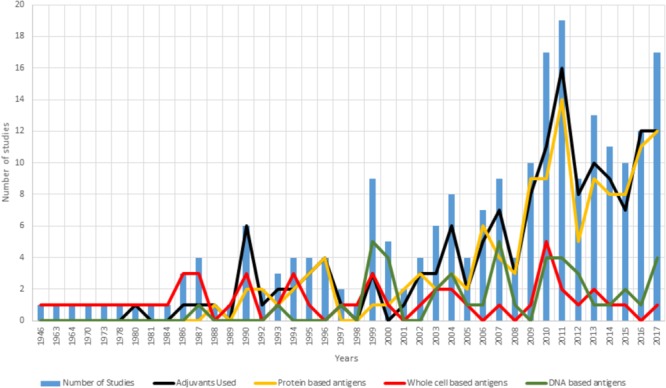
Summary of vaccine formulations between 1946 and 2017. For each year examined, the blue bars represent the number of vaccine trials published that year. Overlayed on the trial numbers is a break down the vaccine formulation tested by inclusion of an adjuvant (black line) and the use of either a protein-based antigen (yellow line), a whole cell antigen (attenuated or in-active) (red line) or a DNA-based antigen (green line).

Over the last 71 years (from 1946 to 2017), there have been a large number of documents (1,489) reported in the scientific literature relating to *Chlamydia* vaccine trials. A literature search of the “Scopus” database using keywords “*Chlamydia* AND vaccine” and limiting the results to full and short communications performed on living hosts to test any form of vaccine targeted toward any species of *Chlamydia* identifies 220 vaccine trials. This represents an incredible body of work that has encompassed different chlamydial species, vaccine formulations (Figure 2) and approaches. Interestingly, the past 10 years have shown the greatest interest in *Chlamydia* vaccine research, with an average of 12 vaccine studies per year. To understand where the *Chlamydia* vaccine field currently stands, it is worth breaking down the 220 trials to evaluate what has been done and how successful they were. All the studies referred to are listed in Supplementary Table S1.

### *Chlamydia* and Host Species Targeted

From the 220 vaccine trials reported, eight chlamydial species and 12 host species have been targeted (Table 2 and Supplementary Table S1): The most targeted chlamydial species, *C. muridarum*, has been studied in 77 vaccine trials (35.0%) using mice (77 trials) and non-human primates (2 trials) as the host. *C. trachomatis* has been used in 67 vaccine trials (30.5%) tested in mice, non-human primates, pigs, guinea pigs, rabbits and a single human vaccine trial. *C. psittaci* has been used in 23 vaccine trials (10.5%) within a range of different hosts, including, mice, sheep, birds, Guinea pigs and cats. Vaccines to *C. pneumoniae* (6.4%) have only been tested in mice, vaccines to *C. pecorum* (5.5%) have been examined in koalas, mice and sheep, vaccines to *C. abortus* (5.0%) have been trialed in mice, cattle, sheep and pigs, vaccines to *C. caviae* (0.5%) have only been tested in guinea pigs and vaccines to *C. felis* (0.5%) have only been trialed in cats. Finally, nine trials (4.1%) have tested vaccines targeted for cross-species protection with a final 5 trials (2.3%) having used the resulting disease name (psittacosis or trachoma) and not a bacterial name as the identifier. This summary reveals that a staggering 85% of vaccine trials have been performed in substitute hosts and is concentrated around developing vaccines for humans. Overall human vaccine trials have been performed in mice, non-human primates, guinea pigs, rabbits and pigs. The only other vaccine trials conducted in a surrogate host were for the development of a sheep vaccine (Table 2). The majority (69.7%) of matched chlamydial species/infected host trials have being conducted within only two species, koalas and sheep. This indicates a major weakness in current *Chlamydia* vaccine research.

### Target Antigens

There have been many different forms of antigen used in vaccine formulation. Traditionally, elementary bodies (EBs) (either live, formalin-fixed, or UV-inactivated) and crude outer membrane preparations were the antigens of choice. More recently (since the 1990’s), antigens have expanded to include the use of recombinant proteins, synthetic peptides, expression vectors and naked DNA.

For the first 40 years of *Chlamydia* vaccine research, the only published antigens used were whole-cell preparations of either live attenuated bacteria (23 studies) or inactivated bacteria (29 studies) (Table 3 and Supplementary Table S1). Inactivation methods for *Chlamydia* EBs consisting of 45% UV, 41% formalin, 10% heat, and 4% other techniques. The first protein-based antigens appeared in 1988 and steadily increased to 130 studies by 2017 (57.7% of all vaccine studies) (Table 3). Despite the successes of whole cell vaccines, safety concerns and the relative costs around the production of a whole cell vaccine has led to significant research being focused on the production of a protein-based vaccine.

**Table 3:** Chlamydial species and the different types of antigens used in trials.

*Chlamydia* species	Antigen type (number of studies)	Total number of studies
*C. abortus*	Plasmid (2), Protein (2), Virus (1), Whole cell (7)	12
*C. caviae*	Protein (1)	1
*C. felis*	Whole cell (1)	1
*C. muridarum*	DNA (1), Nanoparticle (1), Plasmid (5), Protein (63), Virus (1), Whole cell (14)	84
*C. pecorum*	Protein (12), Whole cell (1)	13
*C. pneumoniae*	DNA (1), Plasmid (6), Protein (6), Virus (1), Whole cell (1)	14
*C. psittaci*	Plasmid (3), Protein (7), Virus (1), Whole cell (15)	26
*C. trachomatis*	Nanoparticle (2), Plasmid (16), Protein (43), Virus (7), Whole cell (15)	78

There have been three different types of protein-based vaccines used; 18.5% (24 studies) used crude outer membrane protein preparations, 41.5% (54 studies) used purified recombinant outer membrane proteins and 54.6% (71 studies) used over 143 individual and mixed recombinant and synthetic peptides (Table 3 and Supplementary Table S1). From this array of antigens, MOMP has emerged as the most tested protein (78 trials using MOMP and 71 using other antigenic targets). DNA sequencing and computational analysis has indicated that MOMP is the protein most likely responsible for the success observed in whole cell trials (Stephens et al., 1987; Stephens et al., 1998). The MOMP of *Chlamydia* species is approximately 40 kDa in size, with five genetically conserved domains and four variable domains that are used to determine the serovar within each species (Stephens et al., 1987, 1998; Stevens et al., 2010). These domains contain multiple T-cell and B-cell epitopes that have been shown to induce T-cell immunity and neutralizing antibodies (Caldwell and Perry, 1982; Baehr et al., 1988; Ortiz et al., 1996; Pal et al., 2005). Other chlamydial proteins utilized as antigenic targets have included the polymorphic membrane proteins (PMPs), a collection of surface exposed proteins with highly conserved regions (ideal for inducing cross genotype recognition) and are characterized as autotransporter adhesion molecules involved in early chlamydial infection processes (Vasilevsky et al., 2016). The chlamydial heat shock protein has also been utilized as an antigenic target for multiple trials and has been shown to induce a strong inflammatory response (Morrison, 1991). Another protein tested as an antigenic target has been the chlamydia protease-like activity factor (CPAF), which is a cytosol secreted protein with proposed virulence potential (Li et al., 2007).

In 1987, the first study to use a plasmid-based vaccine was published (Taylor and Prendergast, 1987). This study achieved negative results and no further plasmid-based work was reported for 12 years. In 1999, three groups published five vaccine trials using plasmids, with four showing partial protection (Brunham and Zhang, 1999; Pal et al., 1999; Vanrompay et al., 1999b; Zhang et al., 1999) (Table 3). These trials stimulated *Chlamydia* plasmid-based vaccine research, launching 26 studies over the next 17 years (Table 3 and Supplementary Table S1). There have also been 13 trials (5.5% of vaccine trials) involving naked DNA or adapted virus-based antigens (with 83% of these using either full or partial *omp* gene sequences) occurring sporadically since 1993 (Table 2 and Supplementary Table S1).

### Adjuvants

Within the 220 chlamydial vaccine trials conducted, 73 studies reported no adjuvant present in the vaccine. The majority of these adjuvant-free formulations were for vaccines using either whole-cells or plasmid antigen sources (73%). Of the 147 trials that used an adjuvant, 10 different adjuvants were used in 5 or more distinct trials, with the most utilized adjuvant being CpG oligodeoxynucleotides (all classes) (31%) (Supplementary Table S1). The use of adjuvants in combination with protein-based vaccines (peptides or full-length MOMP) appears to improve vaccine effectiveness, with vaccine trials reporting a 24% increase in achieving partial protection (from 58 to 82%) and a 6% increase in achieving full protection (0 – 6%). Within the *C. pecorum* koala vaccine trials, a triple adjuvant mixture has been trialed with notable success. This adjuvant mixture contains a synthetic host defense peptide IDR-1002, known to be anti-inflammatory (Wu et al., 2017), a synthetic polyphosphazene polyelectrolyte poly [di (sodiumcarboxylatoethylphenoxy) phosphazene] (PCEP), a protein carrier shown to have immune-stimulating properties through acid functionalities (Andrianov et al., 2004) and polycytidylic acid (Poly I:C), a synthetic analog of double stranded ribonucleic acid that is recognized by toll-like receptor 3 (TLR3) and upregulates cytokines involved in Th2 immune responses (Alexopoulou et al., 2001; Matsumoto et al., 2002; Yamamoto et al., 2003; Cheng et al., 2011a). An advantage of this triple adjuvant formulation is that it allows for a single dose vaccine. Sadly, with over 50 different adjuvants having been tried in different vaccine formulations, all having unique mechanisms of action, only limited conclusions can be made by comparison.

### Vaccine Delivery Sites

The methods for vaccine delivery within the published 220 vaccine studies were either systemically, mucosally or a combination of both (Table 4 and Supplementary Table S1). Systemic vaccination has involved 23 studies that used multiple body sites and 107 studies that used a single body site for vaccination, with the most common site being subcutaneous (51.4%), followed by intramuscular (29.9%), intraperitoneal (7.5%), intravenous (4.7%), epidermis (<2%), intra-abdominal (<2%), intradermal (<2%), and transcutaneous (<2%) (Table 4). Mucosal vaccination has involved two studies using multiple mucosal sites and 45 studies have used a single mucosal site, with the most common site being nasal (73.3%), followed by oral (11.1%), ocular (8.9%), vaginal (4.4%), and gastrointestinal (2.2%) (Table 4). Finally, 43 studies have used a combined approach with both systemic and mucosal vaccinations involving sites listed above (Table 4).

**Table 4:** Site for vaccine delivery by chlamydial species and host.

*Chlamydia* species	Mice	Non-human primate	Guinea pig	Rabbit	Pig	Minipig	Bovine	Ovine	Avian	Feline	Koala
**Mucosal**					
*C. abortus*	1							1			
*C. caviae*			1								
*C. muridarum*	19										
*C. pecorum*											1
*C. pneumoniae*	2										
*C. psittaci*			2								
*C. trachomatis*	9	3	1		1						

**Total**	**31**	**3**	**4**	**0**	**1**	**0**	**0**	**1**	**0**	**0**	**1**

**Systemic**					
*C. abortus*	4				2		1	2			
*C. felis*										1	
*C. muridarum*	36										
*C. pecorum*								1			11
*C. pneumoniae*	9										
*C. psittaci*	5							6	3	1	
*C. trachomatis*	32	3		1	1	1					

**Total**	**86**	**3**	**0**	**1**	**3**	**1**	**1**	**9**	**3**	**2**	**11**

**Duel vaccine delivery - Mucosal and Systemic**					
*C. muridarum*	20	2									
*C. pecorum*											
*C. pneumoniae*	3										
*C. psittaci*			1						2		
*C. trachomatis*	10	1			1	1					

**Total**	**33**	**3**	**1**	**0**	**1**	**1**	**0**	**0**	**2**	**0**	**0**

### Vaccine Results, No Challenge

Of the 44 studies that did not include a post-vaccine *Chlamydia* challenge, 18 studies were performed in mice, 11 studies in koalas, four studies in sheep, and one or two in birds, pigs, humans, non-human primates and rabbits. In some of these studies, such as with the human and koala subjects, ethical restrictions most likely prevented the study design from including a challenge component (i.e., deliberate infection). This type of restriction will continue to be a limitation for vaccine design in some hosts and helps explain why the *Chlamydia* mouse model remains a popular research tool. The results of these no challenge trials varied and were dependant on the *in vitro* tests performed on collected samples, predominately focused on *Chlamydia* specific antibody responses (IgG and IgA). However, overall, 86% of the trials achieved results indicating vaccination induced a measurable antibody mediated immune response.

### *Chlamydia* Challenge Trials

Post vaccination studies predominately use challenge trials to test the effectiveness of the trial vaccine, however, as challenge trials are only performed in the short term (<1 year), they only provide information on short term immunity. Observations of long lasting immunity (> 1 year) are lacking in chlamydial vaccine research. From 220 vaccine studies, 176 (80%) employed a *Chlamydia* challenge post-vaccination. These studies involved 159 mucosal challenge routes including urogenital (vaginal, ovarian, uterine, penile, and urethral) (57.1%), nasal (27.3%), ocular (8.7%), oral (0.6%), and multiple sites (4.3%) (Table 5 and Supplementary Table S1). Of the 17 studies that used systemic routes, challenge sites included intraperitoneal (47.1%), subcutaneous (29.4%), intradermal (17.6%), and intra-cerebral (5.9%) (Table 4 and Supplementary Table S1). There are no studies to date that have used a combined mucosal and systemic challenge method. This preference for mucosal-based challenge delivery methods is likely because studies are trying to replicate the natural infection routes of many *Chlamydia* species.

**Table 5:** Challenges based on *Chlamydia* species and anatomical site.

Site	*C. abortus*	*C. caviae*	*C. felis*	*C. muridarum*	*C. pecorum*	*C. pneumoniae*	*C. psittaci*	*C. trachomatis*	Total
Nasal			1	27		13	6	5	52
Urogenital	1	1		52		1	1	50	106
Ocular			1	3			3	8	15
Oral							2		2
Systemic	8				1		8	1	18

**Total**	**9**	**1**	**2**	**82**	**1**	**14**	**20**	**64**	**193**

Of the challenge-based vaccine studies, five different vaccination/challenge methods were implemented; (1) mucosal route of vaccination with a mucosal challenge (41 studies), (2) mucosal route of vaccination with systemic challenge (2 studies), (3) systemic route of vaccination with a mucosal challenge (81 studies), (4) systemic route of vaccination with a systemic challenge (15 studies) and (5) mixed route of vaccination with a mucosal challenge (37 studies). The most common type of vaccine was trials on mice using protein-based vaccines and CpG oligodeoxynucleotides (all classes) adjuvants with 39 trials (16.4%), while the next closest focus was on koalas using a protein-based antigen with 11 trials (5%). From all 176 challenged-based vaccine studies, only 8.5% (15/176) reported complete protection (defined as the absence of pathology and bacterial shedding at challenge site) with 10 of these trials using either a whole cell or MOMP-based antigen (Supplementary Table S1). Interestingly, this group of complete protection trials included three peptide-based studies targeting human *C. pneumoniae* using the mouse model (Tammiruusu et al., 2007; Thorpe et al., 2007; Li et al., 2010). In fact, 66.7% (102 trials) of challenge trials were performed with chlamydial species infecting non-native hosts, meaning that future work will be needed to confirm the efficacy of tested vaccines in their target hosts. Focusing only on studies that matched chlamydial species to infecting host (73 studies), vaccine trials that used a mucosal route of vaccination achieved a measurable mucosal immune response in 80% (20/25) of trials. This compared to systemic vaccination, which showed 77% (37/48) of trials identifying a measurable mucosal immune response.

## Vaccine Trials for Human Chlamydial Infections

The need for a vaccine to manage and reduce human chlamydial disease is well recognized. The prevalence of *C. trachomatis* is estimated at approximately 4.2% among 15 to 49-year-old men and women, making this pathogen the world’s most reported STI (Newman et al., 2015). *C. trachomatis* is known to cause serious urogenital and ocular disease outcomes (Garland et al., 1995; Stevens et al., 2010; Menon et al., 2015). However, with up to 80% of infections asymptomatic, most infections are not recognized and transmission to partners is common (Korenromp et al., 2002; Farley et al., 2003; Ljubin-Sternak and Mestrovic, 2014; Menon et al., 2015). *C. trachomatis* has been reported to have treatment failures (organisms detected after antibiotic treatment has finished) (Jones et al., 1990; Lefevre et al., 1997; Somani et al., 2000; Misiurina et al., 2004; Bhengraj et al., 2010), however no genetic link to macrolide or fluoroquinolone resistance has been reported (Deguchi et al., 2018). Furthermore, chlamydial persistence (organisms present in a non-infectious and non-replicating state) has also been reported in many studies. However, the high rate of individuals being reinfected from undiagnosed partners makes determining whether *Chlamydia* detected after treatment is the result of persistence, treatment failure or reinfection is complex. Recent studies targeting only actively replicating chlamydial cells have demonstrated that a high percentage of treatment failure cases are only identifying inactive cells (via DNA) as false positives for active infections (Janssen et al., 2016; Phillips et al., 2018). The foundational work necessary for developing a *C. trachomatis* vaccine for human use has taken place in mouse and non-human primate model systems. Here we discuss the range of vaccine trials used in mouse model trials, the rationality for progression to new complex vaccines and the relative effectiveness of each vaccine make up.

### Mouse Model Vaccine Trials

*Chlamydia muridarum* is known to infect mice and rats causing conjunctivitis, respiratory disease and urogenital disease. The mouse model has been used as a host for vaccination trials because it is an amenable animal model, enabling post vaccination challenge experiments and detailed analysis of immune responses. The main purpose of studying chlamydial vaccines within the mouse model is for future development of a human chlamydial vaccine. Due to differences in the efficiency of certain strains within the mouse model, many studies chose to use *C. muridarum* as a surrogate for *C. trachomatis*.

Early mouse model trials for *Chlamydia* vaccine research started in the late 1940s and were designed to target *C. psittaci* (known now to be *C. muridarum* post re-classification of nomenclature) infections using live and inactivated whole cell preparation vaccines (Morgan and Wiseman, 1946). These early trials used rates of chlamydial shedding post challenge as a measure of effectiveness and were successful in achieving some protection from bacterial challenge (Morgan and Wiseman, 1946; Mitzel et al., 1970; Johnson and Hobson, 1986; Rekiki et al., 2004b). However, since the 1990s, the predominant focus of mouse model vaccines has been using *C. muridarum* and *C. trachomatis* strains. Live whole cell vaccines have demonstrated the feasibility of inducing a protective response to *Chlamydia* infections. Several trials have shown that using live chlamydial EBs can induce complete protection through intranasal vaccination (Pal et al., 1994; Peterson et al., 1999; Lu et al., 2002). However, further trials have failed to reproduce these results using inactivated EBs, which is thought to be the result of lowered peptide loading on to dendritic cells of inactivated EBs compared to active EBs (De La Maza et al., 2017).

The first MOMP-based vaccine in mice was by Tuffrey et al. (1992). Using a recombinant MOMP (rMOMP) from *C. trachomatis* serovar L1 and direct vaccination into the Peyer’s patches or oviducts, the trial failed to reduce colonization or disease development. However, humoral immune responses in plasma anti-MOMP IgG were detected along with trace levels of mucosal anti-MOMP IgA. This disappointing result was repeated in six additional protein-based vaccine trials over the next 20 years, with the only improvement being that the increases in plasma IgG responses had *Chlamydia* neutralizing effects (Su and Caldwell, 1993; Knight et al., 1995; Igietseme and Murdin, 2000; Shaw et al., 2002; Zheng et al., 2006; Jiang et al., 2015). None of these seven trials used an adjuvant to stimulate immune responses during vaccination, generating a clear indication that an adjuvant-based vaccine would be required for a successful immunological response within a protein-based vaccine.

The first evidence of a protein vaccine inducing protection in mice was by Pal et al. (1997). The vaccine consisted of a detergent prepared chlamydial outer membrane complex (COMC) or denatured MOMP protein in Freund’s incomplete adjuvant. This vaccine, delivered subcutaneously, decreased chlamydial shedding rates and induced protection from disease post-ovarian challenge, with the COMC formulation outperforming the MOMP formulation. The promising results were replicated by Pal et al. (2001) with the MOMP protein, whereby a native MOMP (nMOMP) preparation outperformed the denatured MOMP preparation. Cellular immune responses were enhanced in the nMOMP vaccinated mice and humoral immune responses were slightly higher in the denatured MOMP preparation. These results indicated that conservation of the native protein structure resulted in increased protection from disease and that this is driven by both cellular and humoral immune responses. Pal and colleagues went on to reproduce these results in another three trials testing different adjuvants (CpG and Montanide ISA 720) using nMOMP as the antigen. Furthermore, they also observed similar levels of protection between nMOMP vaccines and live EB vaccines post-intrabursal challenge (Pal et al., 1997, 2005). This was significant, as protein-based vaccines had not performed as well as whole cell vaccines previously. Further mouse model studies into the protective effects of nMOMP have been demonstrated in the respiratory tract (Sun et al., 2009) and the genital tract (Farris et al., 2010; Carmichael et al., 2011; Tifrea et al., 2011). These studies confirm that nMOMP induces protection from disease and decreased bacterial shedding from *Chlamydia* challenge. They also conclude that a cellular and humoral immune response is required for complete protection.

Although native protein vaccine production is less expensive than whole cell vaccine production, antigen in this format is still relatively costly. As such, significant recent research has been focused on the production of a recombinant MOMP (rMOMP) vaccine (where the desired protein antigen is cloned, expressed and purified from *Escherichia coli* in large quantities instead of purified directly from cultured chlamydial cells). Since 1992, there have been 38 different vaccine trials using an rMOMP antigen with a total of 14 different adjuvants. The first reports of a successful rMOMP vaccine was reported by Berry et al. (2004) using a cholera toxin and CpG adjuvant through a transdermal vaccination route (Berry et al., 2004). They observed both humoral and cellular immune responses that included anti-chlamydial plasma and mucosal IgG and mucosal IgA and IFNγ-secreting T cells. Together, these responses reduced chlamydial shedding and enhanced protection against pathology (Berry et al., 2004). Further trials generated comparable results using transdermal, oral and intranasal vaccination routes (Hickey et al., 2004, 2009, 2010). Additional vaccination route studies demonstrated that combining both mucosal and systemic vaccination routes induced an enhanced immune response compared to using a mucosal route alone. Ralli-Jain et al. (2010), Carmichael et al. (2011) and Cheng et al. (2014) all observed that vaccinations with rMOMP and adjuvants CpG and Montanide induced the strongest humoral and cellular immune responses (IgG, IgA and neutralization effect and T-cell proliferation) with a dosage regime of a mucosal priming dose, followed by a systemic boosting dose.

Further insights into the required cellular immune response were observed by O’Meara et al. (2013). They tested an rMOMP antigen with a cholera toxin/CpG adjuvant and various vaccination routes (transdermal, sublingual or intranasal) and observed a relationship between the production of IFNγ, TNFα, and IL17 and protection against infection (O’Meara et al., 2013). Further studies in 2016 (using nMOMP or rMOMP antigens with CAF01 and CAF09 adjuvants) also observed a correlation between an increased production of IFNγ and IL17 and low levels of IL4 with significantly reduced bacterial shedding and protection against respiratory pathology (Pal et al., 2017b).

In the last 3 years, there has been a significant focus on identifying the specific chlamydial outer membrane protein epitopes that induce the highest antigenic response, with a particular focus on the MOMP and PMPs. Pal et al. (2017a) completed one of the largest of these trials, comparing the vaccination effects of nine different PMPs from *C. trachomatis* in mice and challenged with *C. muridarum*. They identified that, although PMPs C, G and H individually elicited the highest levels of humoral and cellular immune responses and decreased signs of disease of the nine different PMP antigens tested, these results were still lower than the more effective *C. muridarum* MOMP vaccine response (Pal et al., 2017a). These results show that the complexities involved with inducing a protective response to chlamydial infections involves the recognition of multiple epitopes. This further indicates that the success of a future chlamydial vaccine will probably be dependent on large recombinant proteins such as the MOMP.

### Non-human Primate Vaccine Trials

One of the most difficult aspects of *Chlamydia* vaccine research is replicating mouse vaccine trial results in other host species, specifically non-human primates. Over the last 30 years, there have been only 12 attempts to replicate mouse model trial results in non-human primates. The first set of non-human primate trials utilized whole cell antigenic targets and observed limited to no protection post challenge (Chang et al., 1964; Whittum-Hudson et al., 1986; Taylor et al., 1987). In fact, one study observed increased chlamydial pathology in vaccinated owl monkeys post ocular challenge (MacDonald et al., 1984). However, recent whole cell antigen vaccines have produced promising results. Kari et al. (2009, 2011) vaccinated Cynomolgus macaques (via the ocular route) with a plasmid deficient strain of *C. trachomatis* and observed increased serum AMI, specifically IgG and IgA, with neutralizing properties, upregulation of both IFNγ and IL12 [from peripheral blood mononuclear cells (PBMC) fractions] and demonstrated protection from ocular challenge in five out of six vaccinated animals (Kari et al., 2009, 2011). These results were repeated in 2014 by the same group, using a systemic vaccination regime (Olivares-Zavaleta et al., 2014). Alternative approaches to reducing EB virulence, such as using a plasmid negative chlamydial strain, failed to induce protection in rhesus macaques (Qu et al., 2015).

Sadly, protein based vaccine trials in non-human primates have shown little to no promise, with six separate trials observing some non-significant increases in both humoral and cell mediated immunity that failed to induce complete protection from disease (Taylor et al., 1987, 1988; Su and Caldwell, 1993; Campos et al., 1995; Kari et al., 2009; Cheng et al., 2011b).

Mouse model trials highlight the complexities involved in producing a vaccine response that will protect from infection and development of disease. Although some success has been achieved through live vaccines and MOMP-based vaccines, failure to reproduce these results in non-human primate models is most likely due to the differences in both host immunity and *Chlamydia* species genetics. Important differences within the tryptophan synthase genes between *C. muridarum* and *C. trachomatis* have the potential to affect how each pathogen will be cleared by IFNγ and IDO pathways in different hosts. Despite this failure, mouse models have demonstrated important characteristics that should be considered in both administration of a *Chlamydia* vaccine and monitoring of the humoral and cellular immune responses. Together, this research identifies that a successful *Chlamydia* vaccine will combine a mucosal and systemic dose regime using a MOMP-based protein with an adjuvant. Success will likely be observed through a combination of (a) humoral immune responses including chlamydial specific plasma IgG and mucosal IgG and IgA responses with neutralizing capabilities and; (b) cellular immune responses, including upregulation of IFNγ, TNFα and IL17 and down regulation of IL4 and IL10.

## Vaccine Trials for Animal Chlamydial Infections

Beyond the quest to develop a vaccine in mice and non-human primates to eventually combat *C. trachomatis* infection in humans, there are several chlamydial species of economic and wildlife welfare importance to merit vaccination programs of their own. So far, concerted effort has been focused on *C. psittaci* and *C. pecorum* for livestock applications and *C. pecorum* for disease management in koalas. To date, only two chlamydial vaccines are available commercially (as whole cell vaccine formulations) and they target *C. felis* in cats and *C. abortus* in sheep. While some of the lessons learnt with *C. trachomatis* and *C. muridarum* in the mouse model can be extrapolated to other chlamydial species and hosts, much work is still needed to tailor specific chlamydial vaccines to their specific purposes.

### Livestock Vaccine Trials – *C. psittaci* Vaccines

Vaccines targeting *C. psittaci* have been trialed sporadically over the last 70 years with 23 studies occurring between 1978 and 2017. However, caution must be taken when comparing early and recent trials, as changes in 1999 to chlamydial nomenclature significantly altering the number of strains included under this once broad species (Everett et al., 1999; Stephens et al., 2009; Greub, 2010).

Of the 18 trials conducted prior to 1999, 67% utilized whole cell preparations as antigenic targets for use in sheep (six trials), guinea pigs (three trials), mice (two trials), and cats (one trial). These trials achieved partial protection from infection and the majority showed reductions in disease (Nichols et al., 1978; Malaty et al., 1981; Johnson and Hobson, 1986; Rodolakis and Souriau, 1986; Wills et al., 1987; Rank et al., 1990; Wilsmore et al., 1990a,b; Gajdosová et al., 1994; Westbay et al., 1994; Jones et al., 1995). There have been three studies utilizing outer membrane preparations (two in sheep and one in mice) and, although the results were promising, the reductions in disease (noted as abortion rates) were lower when compared to that observed from whole cell preparations (46 and 70% reduction, respectively) (Anderson et al., 1990; Tan et al., 1990; Sandbulte et al., 1996). Finally, in 1999, there were two trials conducted in turkeys utilizing a plasmid-based antigenic target and different vaccination routes (systemic and mucosal), however both these trials failed to induce a chlamydial specific antibody response (Vanrompay et al., 1999a,b).

Post 1999, there have been only five trials of *C. psittaci* vaccines. These trials utilized recombinant proteins (three trials) or plasmids in different vectors (eukaryotic and viral) (two trials). Results from all five studies observed partial protection from infection and/or disease; however, each utilized a different gene or protein making comparisons difficult (Loots et al., 2006; Qiu et al., 2010; Liu et al., 2015; Liang et al., 2016; Ran et al., 2017).

### Livestock Vaccine Trials – *C. pecorum* Vaccines

There have only been two *C. pecorum* specific vaccine trials performed in non-koala hosts. Rekiki et al. (2004a), vaccinated mice with a commercial *C. abortus* (temperature-sensitive mutant) live cell vaccine and challenged with *C. pecorum* (sheep isolate) (Rekiki et al., 2004a). They reported some protection (as abortion rates and progeny health) post intraperitoneal challenge, however these pathologies are rarely related to *C. pecorum* infections (Rekiki et al., 2004a). Finally, Desclozeaux et al. (2017a), vaccinated pregnant ewes and lambs with a recombinant PmpG protein from *C. pecorum* strain IPA (sheep isolate) and a rMOMP from a *C. pecorum* genotype G strain (koala isolate) (Desclozeaux et al., 2017a). They observed increases in antibody mediated immunity (IgG and IgA) and cellular immunity (IFNγ), however, *in vitro* neutralization effects were absent and the cell mediated immune response durations appeared short (Desclozeaux et al., 2017a). These studies show some promise in producing an effective *C. pecorum* vaccine. However, as some or all of the antigenic targets utilized, in both these studies, differed to the infecting chlamydial species or strain of interest, these results should be interpreted with caution, with further studies required replicating natural conditions.

### Koala Vaccine Trials

The development of a *C. pecorum* vaccine for koalas has involved a systematic research program that covers 11 different trials over a period of 7 years (Table 6 and Figure 2). The first koala *C. pecorum* vaccination trial tested the safety and immunogenicity of recombinant forms of *C. muridarum* proteins including MOMP, Ribonucleotide reductase (NrdB) and *omp*85 (CT0512), as well as adjuvant effects of Immunostimulating complex (ISC), Aluminum hydroxide gel (Alhydrogel) and TiterMax Gold (Carey et al., 2010). This first study vaccinated healthy captive female koalas, utilizing a three-dose vaccination regime and tested the effects of each adjuvant over a 270 days period. The results identified strong neutralizing (*C. muridarum* and *C. pneumoniae*) plasma derived IgG responses lasting for > 270 days using the ISC and Alhydrogel and cloacal IgG responses lasting for > 270 days in the ISC group (Carey et al., 2010). This study demonstrated that a *Chlamydia* based vaccine in koalas was possible and induced a significant increase in plasma and mucosal. While the results of the first trial were promising, they utilized antigens from *C. muridarum*, the mouse pathogen, rather than from the koala pathogen, *C. pecorum*. The next vaccine trial replaced *C. muridarum* rMOMP (genotype G) and NrdB with *C. pecorum* proteins and containing only the adjuvant ISC. This trial included koalas with signs of clinical disease (conjunctivitis and/or cystitis) and a group of healthy koalas receiving only two vaccine doses (all other groups received a three dose vaccination) (Kollipara et al., 2012). This study demonstrated similar plasma and mucosal IgG responses as in Carey et al. (2010) for both MOMP and NrdB, with MOMP eliciting a slightly higher elevation of the humoral immune response compared to NrdB. Interestingly, koalas with current signs of disease showed some clinical signs of improvement post vaccination and the two dose vaccination regime resulted in similar IgG levels as the three dose regime. The success of a koala specific *C. pecorum* targeted vaccine lead to three additional trials that (1) expanded the vaccine to be multi-variant by including multiple MOMP genotypes within a single vaccine to induce a broad antigenic memory (Kollipara et al., 2013b), (2) observed plasma antibodies derived from koalas immunized with rMOMP produced antibodies to both variable and conserved domains of MOMP (Kollipara et al., 2013a), and 3) found that different vaccination routes (mucosal via intranasal or systemic via sub-cutaneous) had differing affects (Waugh et al., 2015). Collectively, the koala specific *C. pecorum* vaccine trials indicated that a multivalent rMOMP vaccine, delivered via a subcutaneous and intranasal route, could elicit a cross-protective humoral and cellular immune response in wild koalas, with or without current *Chlamydia* infections and related signs of disease.

**Table 6:** *Chlamydia* vaccine trials in koalas.

Antigen	Adjuvant	Dose	Koalas	Captive/Wild	Results	Reference
MOMP, NrdB and omp85^a^	ISC, Alhydrogel, TiterMax	3	18 healthy females	Captive	ISC performed the best with mucosal > 270 IgG	Carey et al., 2010
MOMP, NrdB	ISC	2, 3	12 Healthy, 12 Diseased male and female	Wild	Increase in IgG in both health and diseased koalas and between 2 and 3 dose regimes	Kollipara et al., 2012
MOMP A, F and G	ISC	3	12 Healthy females	Captive	Plasma and mucosal IgG homologous and heterologous recognition of MOMP types	Kollipara et al., 2013b
MOMP A, F, and G	ISC	3	5 diseased, 4 Healthy male and female	Captive and Wild	Vaccination induced greater epitope recognition compared to natural infection (including conserved regions)	Kollipara et al., 2013a
MOMP	ISC	3	12 Healthy males	Captive	Intranasal increased humoral immune response subcutaneous increased CMI responses	Waugh et al., 2015
MOMP A, F, and G	ISC	3	60 healthy male and female	Wild	Decreased *C. pecorum* load and disease prevalence in vaccinated free-range koalas	Waugh et al., 2016b
MOMP	Not stated	3	20 Healthy male and female	Wild	Increased *C. pecorum* IgG neutralization effect elicited by the vaccine	Khan et al., 2016b
MOMP A, F, and G	Tri-Adj	1, 2	6 healthy females	Captive	Comparable humoral/cellular immune responses in both single and double dose regimes	Khan et al., 2014
MOMP A, F, and G	ISC, Tri-Adj	1, 3	15 healthy male and female	Wild	Establishes a basis for the use of a 1 dose vaccine that can induce comparable and enhanced immunological responses compared to a 3-dose vaccine.	Khan et al., 2016a
MOMP A, F, and G	Tri-Adj	1	6 disease sex not stated	Wild	Decreased conjunctival pathology and *C. pecorum* DNA shedding in both vaccine and antibiotic treated koalas	Waugh et al., 2016a
MOMP A, F, and G PmpG	Tri-Adj	1	63 healthy male and female	Wild	Comparable results between antigens. However, some development of disease post vaccination	Desclozeaux et al., 2017b

*^*a*^Using proteins derived from *C. muridarum* and C. trachomatis.*

With previous trials observing strong immune responses to vaccination, further studies aimed to determine the vaccines ability to limit disease progression in wild koalas over extended periods (up to 12 months) (Khan et al., 2016b; Waugh et al., 2016b). Over two studies, koalas vaccinated with two doses of an rMOMP mixed genotype *C. pecorum* vaccine containing ISC adjuvant and were followed for 12 months. Vaccinated koalas had decreases in both *C. pecorum* infecting loads and disease prevalence at both six and 12 months post vaccination, as well as showing a significant increase in plasma IgG that recognized epitopes within the conserved domains of *C. pecorum* 6 months post vaccination (Waugh et al., 2016b). When compared to unvaccinated controls, naturally infected vaccinated koalas also had a significant increase in the neutralizing effect of the plasma derived immunoglobulins (Khan et al., 2016b).

One major drawback to the *C. pecorum* koala vaccine as it was formulated to date was that it required multiple vaccine doses for the ISC adjuvant to induce optimal protection. Given the challenge of a multiple vaccination strategy in a wildlife management program, a redesign of the adjuvant component was undertaken to allow for single dose implementation. In 2014, the mixed genotype rMOMPs were combined with a new adjuvant known as Tri-Adj, developed from the Veterinary Infectious Disease Organization (VIDO), Canada (Khan et al., 2014; Waugh et al., 2016a). The Tri-Adj combines three different components; a synthetic host defense peptide IDR-1002 (known to be anti-inflammatory; Wu et al., 2017), a synthetic polyphosphazene polyelectrolyte poly [di (sodiumcarboxylatoethylphenoxy) phosphazene] (PCEP) [a protein carrier shown to have immune-stimulating properties through acid functionalities (Andrianov et al., 2004)] and polycytidylic acid (Poly I:C) [a synthetic analog of double stranded ribonucleic acid that is recognized by toll-like receptor 3 (TLR3) and upregulates cytokines involved in Th2 immune responses (Alexopoulou et al., 2001; Matsumoto et al., 2002; Yamamoto et al., 2003; Cheng et al., 2011a)]. This new vaccine formulation was tested using a one and two dose regime with healthy koalas (Khan et al., 2014). Overall, comparable humoral and cellular immune responses were observed in both the single and double dose regimes, indicating the efficacy of a single dose *C. pecorum* vaccine in healthy koalas. Once a single dose regime was established, Khan et al. (2016a) sought to compare specific humoral and cellular immune responses between the old three dose ISC adjuvant regime and the new Tri-Adj single dose regime on free-ranging koalas using the mixed genotype rMOMP antigens. Humoral immune responses (plasma IgG) observed between the two groups showed an increased plasma IgG response from the three dose ISC vaccine group, however, when assessed for neutralizing effects on *C. pecorum* EBs, there were no differences observed between vaccine groups. As well, there were major differences in the *C. pecorum* epitopes recognized by plasma IgG from the ISC vaccine and the Tri-Adj vaccine group. The Tri-Adj vaccine group recognized two extra epitopes with a conserved domain and one extra epitope within variable domain compared to the ISC vaccine group. Collectively, these results solidified the advantages of the new Tri-Adj vaccine formulation over the old ISC vaccine formulation.

With the establishment of a single dose vaccine, the most recent trials have aimed at replicating the vaccine’s effectiveness on koalas with current signs of chlamydial disease over an extended time period (6 months) (Waugh et al., 2016a; Desclozeaux et al., 2017b) and testing the utility of expanding the antigenic targets included in the formulation to include PMPs along with rMOMPs (Desclozeaux et al., 2017b). The single dose rMOMP/Tri-Adj vaccine reduced the severity of the conjunctival pathology and *C. pecorum* DNA shedding in both vaccinated and antibiotic treated koalas and elicited a long lasting response to *C. pecorum* stimulus (*in vitro*) (Waugh et al., 2016a; Desclozeaux et al., 2017b). However, lasting protection from *C. pecorum* reinfection appeared elusive, as both rMOMP and rMOMP/PMP vaccine groups contained koalas with signs of chlamydial disease at a prevalence similar to unvaccinated controls. Further analysis of these *C. pecorum* strains indicated that these strains contained a large genetic diversity within the variable domain four of the *ompA* gene, known to contain T and B cell epitopes. Their conclusions noted that these observations indicate a possible pitfall in using a single antigenic region (MOMP) and that considerations should be made to develop a multi-antigenic vaccine with the possibility of including a mucosal administration site in combination with the current sub-cutaneous route.

This systematic series of vaccine trials identifies that with direction and persistence an effective chlamydial vaccine can be achieved within the intended host. Future directions for the koala vaccine may include alterations to vaccine formulations used, such as a second vaccine dose at a mucosal site, but with the foundation of work already completed the effects of the alterations of vaccine effectiveness can be accurately assessed.

## Conclusion

This review has identified that, in over 70 years of vaccine research, with many advances in techniques and knowledge of the target species, no single antigen type or target, adjuvant, or route of administration has been established as a clear front-runner for effective vaccination. Extensive mouse model trials indicate that whole cell antigenic targets induce an effective response, protecting from disease and reducing shedding rates. However, replication of these results using more commercially acceptable antigenic preparations has proven difficult. Both mouse and koala trials indicate that MOMP is a highly recognized antigenic target and is a suitable substitute for whole cell targets. However, if MOMP is not combined with an appropriate adjuvant, it is ineffective. There is also evidence indicating a combined systemic and mucosal vaccine delivery is very effective, however, this is likely to depend on the target species, host and adjuvant used. Mouse model trials have been important in understanding the humoral and cellular immune responses required for an effective chlamydial vaccine, unfortunately replication of these responses has failed in larger animals including humans. These failures are likely due to the inherent differences in chlamydial – host interactions, such as IFNγ induction of p47 GTPase in mice and IDO in humans (Bonner et al., 2014).

With future trials utilizing closer related host species (i.e., non-human primates) with focus on the differences in these interactions and specific adjuvant combinations, it is plausible that an effective human vaccine is possible. With the first human phase 1 clinical trials currently underway, this establishes a major milestone for chlamydial vaccine development and will provide answers to many questions related to the effectiveness of the vaccine within the intended host.

## Data Availability Statement

All datasets for this study are included in the manuscript and the Supplementary Files.

## Author Contributions

SP, BQ, and PT contributed to the conceptualization. SP performed the data curation and analysis. SP, BQ, and PT contributed to construction and writing of the manuscript.

## Conflict of Interest Statement

The authors declare that the research was conducted in the absence of any commercial or financial relationships that could be construed as a potential conflict of interest.

## Supplementary Material

TABLE S1 List of all data collected from all trials included in analysis with associated references.
