# Supplementary material for: Seventy Years of Chlamydia Vaccine Research – Limitations of the Past and Directions for the Future
Source: Front Microbiol. 2019 Jan 31;10:70. doi: 10.3389/fmicb.2019.00070 (PMC6365973; doi:10.3389/fmicb.2019.00070)
Supplement: TABLE S1 — List of all data collected from all trials included in analysis with associated references. [file Table_1.DOCX]

| **Chlamydia species** | **Year** | **Antigen type** | **Adjuvant** | **Host** | **Delivery method** | **Results** | **Notes** | **Ref** |
| --- | --- | --- | --- | --- | --- | --- | --- | --- |
| *Psittacosis* | 1946 | Formalin inactivated | None | Mice | IV | 45% increase in survival rates | Challenge trial Intraperitoneal | [1] |
| *Trachoma* | 1963 | Formalin killed | None | Humans | IV | 2-fold rise in antibody levels |  | [2] |
| *Trachoma* | 1964 | Live attenuated and Formalin inactivated | None | *Macaca rhesus* monkeys | SC | Decreased severity (stronger for live vaccine). | Challenge trial Ocular | [3] |
| *Psittacosis* | 1970 | Formalin inactivated | None | Mice | IP | some degree of cross protection | Cross immunity between strains | [4] |
| *C. abortus* | 1973 | Heat inactivated | None | Bovine | SC and IM | Achieved protection to intradermal challenge but not IM challenge |  | [5] |
| *C. psittaci* | 1978 | Living and Formalin inactivated | None | Guinea pigs | Oral | Decreased severity at both sites for both vaccines | Challenge trial Ocular and Vaginal | [6] |
| *C. trachomatis and C. psittaci* | 1980 | Betaprone-inactivated | complete Freund adjuvant | Guinea pigs | IM (feet) | some degree of cross protection, delayed hypersensitivity, no antibody responses | Testing cell mediated immunity | [7] |
| *C. psittaci* | 1981 | Live | None | Guinea pigs | Ocular | serum and tear antibody levels correlated with resistance to the high dose inoculation | Challenge trial Ocular | [8] |
| *C. trachomatis* | 1984 | ^60^Co irradiation Inactivated and lyophilized | None | Owl Monkeys | Ocular | Ocular and serum Abs increased post vaccination. Upon challenge, vaccinated monkeys developed increased pathology | Challenge trial Ocular – **adverse effects** | [9] |
| *C. psittaci* | 1986 | Live and Formalin inactivated | complete Freund adjuvant | Mice | SC | Live vaccination showed resistance to challenge. Formalin inactivated showed no protection | Intracerebral challenge | [10] |
| *C. trachomatis* | 1986 | Live and UV or Formalin inactivated | None | Cynomolgus monkeys | Oral | Vaccine showed a shift in T-cell populations. Live vaccine had increased IgA post challenge | Ocular challenge | [11] |
| *C. psittaci* | 1986 | Live temperature sensitive strains | None | Goats | ?? | 1 year post vaccination challenge resulted in 80% reduction in aborted pregnancies | Intradermal challenge | [12] |
| *Trachomatis* | 1987 | Recombinant expressing LPS (e.coli) | None | Cynomolgus monkeys | Oral | No induced protection in either IgG or IgA | Ocular Challenge | [13] |
| *C. trachomatis* | 1987 | Live and UV or Formalin inactivated | Freund's adjuvant | Cynomolgus monkeys | Oral via gastric tube, Rectal and IM | Increased IgG and IgA to live vaccine but this did not result in increased protection to challenge compared to natural infection | Ocular challenge to observe differences in delivery method and antigen type | [14] |
| *C. abortus* | 1987 | Live temperature sensitive strains | None | Mice | SC | No differences were observed when compared to single vaccine, indicating no interference of the coxiella vaccine | IP challenge of Abortus to test any differences when the Abortus vaccine is administered with a coxiella vaccine | [15] |
| *C. psittaci* | 1987 | Commercial modified live vaccine | None | Feline (cats) | SC | Decrease in severity of disease compared to non-vaccinated. Also observed prolonged gut sheading post challenge | Ocular challenge trial | [16] |
| *C. trachomatis (MoPn)* | 1988 | SDS extracted MOMP (serovar B) | Cholera toxin (oral only) | Cynomolgus monkeys | Ocular, oral and IP | Little difference between vaccinated and control animals | Ocular challenge trial | [17] |
| *C. trachomatis (MoPn)* | 1989 | Live EBs (LGV) and Chlamydial antiserum | None | Mice (BALB/c) | Esophagus, and IV | Total protection against chlamydial lung disease via oral vaccination | Intranasal challenge | [18] |
| *C. psittaci* | 1990 | Live and UV inactivated | Freund incomplete adjuvant (2xbooster inoculations only) | Guinea pigs | IV, SC, Oral and ocular | Inactive antigen showed decreased disease severity upon challenge | Intravaginal challenge | [19] |
| *C. psittaci* | 1990 | Outer membrane preparation | aluminum hydroxide | Lambs | SC | 46% decrease in abortion rates | SC challenge | [20] |
| *C. psittaci* | 1990 | Outer membrane preparation | aluminum hydroxide | Scottish blackface ewes | SC | No abortions in vaccinated 4/7 abortions from control | SC challenge | [21] |
| *C. psittaci* | 1990 | Inactivated EBs | Yes (not noted in abstract) | Ewes | SC | No shedding or abortions. Healthier lambs (by weight) | SC challenge | [22] |
| *C. psittaci* | 1990 | formalin-inactivated EBs (commercial vaccine A22) | Freund's complete adjuvant | Ewes | SC | Offered poor protection to both A22 and BS strain (slightly better protection to the less virulent A22 strain) | Intranasal and oral challenge | [23] |
| *C. psittaci* | 1990 | Inactivated EBs | None | Ewes | SC | The vaccine was at least 80% efficacious against Chlamydia and Campylobacter spp and appeared to be protective. |  | [24] |
| *C. trachomatis* | 1992 | rMOMP | None | Mice | SC, IM or IP. Oral, directly onto Peyers patches | Reduced Pathology by 77%, but failed to reduce shedding. Produced high levels of circulating anti-rMOMP IgG but only traces of anti-rMOMP IgA in vaginal secretions |  | [25] |
| *C. trachomatis* | 1992 | 30-amino-acid peptide from VD4 of the MOMP | keyhole limpet hemocyanin and Freund’s complete and incomplete adjuvant | Mice | IP | In-vitro sera was cross-reactive with 15 different CT serovars. |  | [26] |
| *C. trachomatis* | 1993 | Live EBs (3 doses) | None | Rat | Ocular, sub-conjunctival, gastrointestinal and IP | Ocular showed the highest mucosal IgA response. | Testing the different administration routes and antibody fluctuations over 3 doses | [27] |
| *C. trachomatis* | 1993 | Oligopeptide sequence to 2 different sections of MOMP (T-Helper and VDIV) | None | H-2 congenic mouse strains and Cynomolgus monkeys | IP – mice  IM - monkeys | Similar results were seen in both hosts.  High-titre IgG to peptide and CT. Also made antibodies to a neutralizing epitope LNPTIAG within the VDIV region of MOMP. There was also evidence of cross protection across different serovars. |  | [28] |
| *C. trachomatis* | 1993 | An adapted poliovirus to contain the VD1 from MOMP. (3 doses) | Freund’s complete and incomplete adjuvant | Rabbits | SC and IM | Induced a strong response against both PV1-M and C. trachomatis serovar A. These response were 10-100 fold higher than nMOMP responses. Also was found to be neutralizing in-vitro and in-vivo (Cynomolgus monkeys conjunctival) | Trial to test a new polio virus MOMP hybrid vaccine in rabbits | [29] |
| *C. trachomatis* | 1994 | synthetic chimeric peptide representing the VD1 and 4 of MOMP (3 doses) | Freund’s complete and incomplete adjuvant | A/J(H-2a), DBA/1(H-2q), C57BL/10(H-2b), CBA/J(H-2k), Balb/c(H-2d) mice | IP | There was a spectrum of antibody responses which ranged from high ELISA and IFA titres by the C57BL/10 mice to little or no response by Balb/c mice. Antisera from C57BL/10 mice recognized all 15 serovars of CT. By combining the antigen with KLH the response by Balb/c and the DBA/1 mice was greatly improved. |  | [30] |
| *C. trachomatis (MoPn)* | 1994 | Live EBs (1 dose) | None | Female BALB/c mice | IN | Increased serum IgG, IgM, IgA and neutralizing antibodies and mucosal IgG and IgA. Also showed complete protection post challenge with greatly reduced pathology and a complete retention of fertility. | Ovarian bursa challenge | [31] |
| *C. psittaci* | 1994 | Formalin-inactivated EBs (1 and 2 doses) | None | Sheep and mice | SC | Significantly reduced lung infections in Mice.  Sheep antibody responses were sporadic for the Complement fixation test but consistently positive using ELISA. | Intranasal challenge (mice).  Humoral immune response in sheep and mice | [32] |
| *C. psittaci* | 1994 | Live EBs or rMOMP | Freund’s complete and incomplete adjuvant | BALB/c and C3H/HeJ  mice | SC | Showed that there are links between MHC complexes and MOMP specific antibody responses. They showed that although both groups of mice produced MOMP specific IgG the mice with different a MCH complex were unable to help the MOMP-specific B cells. | A trial to study the effects of MHC complexes on MOMP antibody responses | [33] |
| *C. trachomatis (MoPn)* | 1995 | Peptide A8-VDIV (antignically common Th and B-cell epitopes of MOMP) | aluminum hydroxide gel or Rehydragel LV | Female A/J Mice | SC | Increased mucosal IgG, no mucosal IgA. | Intravaginal and intrauterine (via injection into the uterine horn)challenge | [34] |
| *C. trachomatis (MoPn)* | 1995 | Synthetic Peptide - 12 amino acid sequence located on the edge of the third variable region (VSIII) of MOMP, | None | C3H and BALB/c mice | intradermal | No increase in antibodies, T cell responses in vaccinated animals, decrease in disease progression but still had some associated uterine inflammation. | Immunogenicity study and uterine cavity challenge | [35] |
| *C. trachomatis (MoPn)* | 1995 | octyl-β-D glucopyranoside extracted MOMP C. trachomatis (serovar C) | Cholera toxin (oral/ocular) complete Freund's adjuvant (IM) | Cynomolgus monkey | Oral, ocular and IM | Increased IgG post vaccination. No significant reduction in disease post challenge. | Ocular challenge | [36] |
| *C. Psittaci* | 1995 | sodium lauryl sarcosine inactivated | ISCOM matrix, Novasomes, Marcol 52/Arlacel A and Alhydrogel | Scottish Blackface X Swaledale ewes and Shetland X Lleyn ewes | SC | Marcol 52/Arlacel A resulted in abscess formation  The best formulation was 16µg antigen with ISCOM matrix at >1 dose, reducing abortion rates from 31.5 to 4.8% and Chlamydia shedding 74 to 24%. | SC challenge to compare adjuvant | [37] |
| *C. Psittaci* | 1996 | MOMP and LPS preparations | Alhydrogel | Female, Swiss White CF-1 mice | SC | Results suggest that MOMP is the major protective antigen compared to LPS | IP challenge | [38] |
| *C. trachomatis (MoPn)* | 1996 | 2x MOMP VD1 and 4 representative peptides (VD1/4 and VD4/1) | complete Freund's adjuvant | C57BL10 Mice | IP | Showed that the position of the epitopes greatly influenced the immune response with in vitro neutralization stronger for VD1/4 | In vitro challenge of serum | [39] |
| *C. trachomatis (MoPn)* | 1996 | Monoclonal anti-idiotypic antibody (Soluble and encapsulated) (targets chlamydia exoglycolipid antigen) | Alumina (oral only) none for SC | BALB/c mice | Oral and SC | Showed pre challenge anti chlamydia antibodies and showed protection from ocular challenge. | Ocular challenge | [40] |
| *C. trachomatis (MoPn)* | 1996 | sodium dodecyl sulfate, n-octyl-b-Dglucopyranoside, and Zwittergent 3-14 derived MOMP and COMC extractions | complete and incomplete Freund’s adjuvant and 0.3% aluminium hydroxide (COMC only) | BALB/c female mice | SC | A significant humoral and cell-mediated immune response was observed in all groups. VD1 abs for all VD3/4 abs from Z3-14 and COMC only. Also COMC had much higher ab levels (including IgA). COMC decreased infection and disease by 58% | Intrabursal (Ovarian) | [41] |
| *C. psittaci* | 1997 | Live vaccine of C. psittaci with a commercial toxoplasma vaccine | None | Scottish blackface ewes | SC | 70% reduction in abortion rates. Also noted a reduction in shedding and disease severity | Day 70 gestation challenge plus combination vaccine compatibility | [42] |
| *C. trachomatis (MoPn)* | 1997 | CTP and MOMP DNA vaccine and live EBs | None | Female BALB/c mice | IM(plasmid)  IP(Live EBs) | MOMP DNA generated significant delayed-type hypersensitivity and serum abs. Also reduced shedding post challenge by >100-fold (CTP had no change) | Intranasal challenge | [43] |
| *C. trachomatis (MoPn)* | 1998 | chlamydial-pulsed DC | None | Female C57BL/10 mice | IV by infusion into the retro-orbital sinus | Immunized mice shed 3 logs fewer infectious chlamydiae, also protection from disease | Intravaginal challenge | [44] |
| *C. trachomatis (MoPn)* | 1999 | DNA plasmid with MOMP insert | None | BALB/cAnN , C3H/ HeN and C57BL/6N mice | IM or IN | No protection from infection or disease | Intravaginal challenge | [45] |
| *C. trachomatis (MoPn)* | 1999 | DNA plasmid with MOMP insert | None | Mice | IM | Developed antigen-specific delayed-type hypersensitivity, lymphocyte proliferation and interferon-γ (IFN-γ) production. IgA increase in 2/3. | Cellular and humoral immune response characterization via nasal challenge | [46] |
| *C. trachomatis (MoPn)* | 1999 | Live or UV inactivated | incomplete Freund’s adjuvant | BALB/c mice | IP | Live vaccine only, increased DTH, INFg and IgA, also had detection of IL-10 and granulocyte-macrophage colony-stimulating factor and enrichment of DCs | Cellular and humoral immune response characterization via nasal challenge | [47] |
| *C. trachomatis (MoPn)* | 1999 | MOMP VD4 peptide | Monophosphoryl lipid A | C3H and C57BL/6 mice | IM with nasal boosters | By combining the MOMP VD4 peptide with a *Chlamdia* heat shock peptide a slightly increased immune response was achieved in C3H mice only | Vaccine was encapsulated with *Chlamydia* heat shock protein, T-cell helper peptide from tetanus toxin abd the pre-S2 region of Hepatitis B virus | [48] |
| *C. psittaci* | 1999 | DNA plasmid with MOMP insert coated in gold beads | None | SPF Turkey | Epidermis via Particle-mediated (gene gun) transfer. | Primed T helper and B cells but failed to induce a high-titer antibody response | Intranasal challenge | [49] |
| *C. trachomatis (MoPn)* | 1999 | Live (1 dose) and UV inactivated (3 doses) | None | C3H/HeJ mice | IN or IP | Live mucosal vaccine showed p<0.05 protection with increased mucosal IgG and IgA, UV inactivated showed no protection | Vaginal challenge | [50] |
| *C. psittaci* | 1999 | DNA plasmid with MOMP insert | None | SPF turkey | IM and IN | Primed T helper and B cells but failed to induce a high-titer antibody response. Did show improved clinical signs | Intranasal challenge | [51] |
| *C. trachomatis (MoPn)* | 1999 | UV-inactivated EBs or RBs (3 doses) | Freund's adjuvant complete (dose 1) and incomplete (dose 2 & 3) | BALB/c mice | SC and IM | No significant differences were noted between tests and controls. Although RB vaccines showed a lowered fertility rate compared to controls. | Ovarian bursa challenge and measurements of fertility rates between vaccines | [52] |
| *C. trachomatis (MoPn)* | 1999 | MOMP DNA or transfected with MOMP, Salmonella typhimurium (3 - 4 doses) | None | BALB/c mice | IM and IN (DNA) or oral transfected with MOMP Salmonella typhimurium | All vaccines showed protection against challenge. | Intranasal challenge | [53] |
| *C. pneumoniae* | 2000 | DNA plasmid with heat shock gene (hsp-60) | None | C57Bl/6 mice | IN and ID | 5–20-fold reduction of *C. pneumoniae* in lungs and increased INF gamma for IN vaccination. ID vaccination showed no protection | Intranasal challenge | [54] |
| *C. trachomatis (MoPn)* | 2000 | DNA plasmid with MOMP insert with immune-stimulating  complexes (ISCOM) of MOMP protein (3 doses) | None | BALB/c mice | IM | Vaccination with follow up ISCOM increased INF gamma and IgA resulting in increased protection | Intranasal challenge | [55] |
| *C. pneumoniae* | 2000 | DNA containing the ORFs for MOMP, ytfF, GltL, pml116, Npt1Cp, DnaK, ndk and dagA | None | BALB/c mice | IN or IM | Only MOMP and Npt1cp showed increased protection to challenge | Intranasal challenge | [56] |
| *C. trachomatis (serovar D)* | 2000 | MOMP preparation with immune-stimulating  complexes (ISCOM) of MOMP protein (3 doses) or Live EBs or UV-inactivated EBs | None | BALB/c mice | IN – All  IM – MOMP-ISCOM (only) | IM – MOMP-ISCOM induced a Th1 response (increased INF-gamma and IL-4) and cleared challenge infections. IN-MOMP-ISCOM only partially cleared challenge infection. | Intravaginal challenge | [57] |
| *C. pneumoniae* | 2000 | MOMP Omp2 or Hsp60 DNA (3 doses) | None | BALB/c mice | IM | pmomp or phsp60 showed 1.2–1.5 log reduction in the mean lung bacterial counts post challenge. No effect on symptoms | Intranasal challenge | [58] |
| *C. trachomatis (serovar K)* | 2001 | monoclonal anti-idiotypic antibody - molecular mimic of the chlamydial glycolipid exoantigen (GLXA) (2 doses) | None | C3H/HeJ mice or  BALB/c mice | Oral, IN or SC | Decreased shedding was observed in all vaccinations. | Intravaginal challenge | [59] |
| *C. trachomatis (MoPn)* | 2001 | Re-folded and glutaraldehyde-fixed MOMP (3 doses) | Complete Freund’s adjuvant – dose 1 incomplete Freund’s adjuvant – dose 2 & 3 | BALB/c female mice | IM and SC | Vortex MOMP preparation showed strong humoral and cell mediated immune response, with les shedding post challenge. Sonicated MOMP preparation had strong humoral but weak cell mediated immune response | Left ovarian bursa challenge | [60] |
| *C. trachomatis (MoPn)* | 2002 | DC pulsed rMOMP (2 doses) | None | Female C57BL/10 | IV | DC pulsed ex vivo with rMOMP generated a Th2 anti-MOMP immune response, and were not protected following infectious challenge. | Intravaginal challenge | [61] |
| *C. trachomatis (MoPn)* | 2002 | MOMP (1, 2 or 3 doses) | CpG suspended in aluminum hydroxide | BALB/c mice | IM or SC | MOMP, CpG, and alum had a high titer of serum antibodies significant lymph proliferation of T-cells and showed significantly less body weight loss. | Intranasal challenge | [62] |
| *C. trachomatis (MoPn)* | 2002 | UV inactivated EBs (2 doses) | Intranasal gene transfer for adenoviral GM-CSF cDNA | Female BALB/c mice | Intranasal (IN) and intrapulmonary (IP) | Mice with the GM-CSF transfer at the site of challenge had increased DCs and lung Ag-specific IgA levels and enhanced Ag-specific CD4-Th1 immune responses, which resulted in protection. | Intranasal challenge – no protection observed when gene transfer and challenge differed in site even with increased systemic immune responses. | [63] |
| *C. pneumoniae* | 2002 | OMP-2, MOMP, HSP-60 (3 doses) | Freund's adjuvant complete (FCA) and incomplete (FIA), also FIA with CpG | C57Bl/6 mice | SC | OMP2/FCA and HSP60/FCA both resulted in adverse effects due to IL-10. These adverse effects were reversed when replaced with FIA/CpG. | Adjuvant/Ag comparison under intranasal challenge | [64] |
| *C. trachomatis (serovar D)* | 2003 | Pgp3 DNA plasmid (3 doses) | None | C3H/HeN mice | Intradermal | Vaccine prevented salpinx infection in 56% of mice (p<0.001) | Vaginal challenge to asses chlamydial assending ability | [65] |
| *C. trachomatis (MoPn)* | 2003 | MOMP prep | Borrelia burgdorferi outer surface protein A (OspA) | C3H/HeN and BALB/c mice | IM, SC, IN, perivaginal and periscular | Significant protection against infection was achieved in the C3H/HeN mice inoculated i.m.+s.c. with MOMP+OspA. | Left ovarian bursa challenge | [66] |
| *C. trachomatis (serovar D)* | 2003 | MOMP plasmid in ghost vibrio cell (3 doses) | Recombinant  *Vibrio cholerae* ghosts | Female BALB/c mice | IM | Immunized mice produced mucosal and systemic Th1 responses. T-cell immunized mice produced partial protection to vaginal challenge. | T-cells from immunized mice were transferred to native mice and then intravaginal challenged | [67] |
| *C. abortus* | 2003 | Formalin inactivated EBs (2 doses) | None | Sows | IM | Increased primary and secondary IgG serum antibodies that had an in-vitro (western blot) response to C. abortus, C. pecorum and C. suis | Serological responses | [68] |
| *C. trachomatis (MoPn)* | 2003 | Live EBs | None | C3H/HeN, BALB/c and C57BL/6 mice | IN | Strong Chlamydia-specific humoral and cell-mediated immunity was detected in all strains. Increased fertility was also shown. | Genital challenge | [69] |
| *C. abortus* | 2003 | *C. abortus* antigen preparation (2 doses) | Aluminium hydroxide, rehydragel, RIBI Adjuvant system Quil A (QA) Specol (Sp), QS-21 (QS), Montanide ISA 206 and 773. | CBA/J and Swiss OF1 mice | SC | QS-21 (QS) or Montanide 773 (M7) induced the best protection both against homologous and heterologous challenge, with an adequate (Th1-like) immune response | Intraperitoneal challenge | [70] |
| *C. muridarum* (re-classified from *C. trachomatis (MoPn))* | 2004 | Isolated recombinant MOMP protein (4 doses) | cholera toxin and CpG oligodeoxynucleotides | BALB/c mice | Transcutaneous | MOMP-specific IgG and IgA in vaginal and uterine lavage  Fluid, and MOMP-specific IgG in serum, and gamma interferon-secreting T cells in reproductive tract-draining caudal and lumbar lymph nodes, and enhanced clearance. | Intravaginal challenge | [71] |
| *C. pecorum and C. abortus* | 2004 | Live EBs of *C. abortus 1B* | None | OF1 mice | SC | *C. pecorum* – 100-fold reduction in PFU although *C. pecorum* has lowered virulence in mice compared to *C. abortus* | Intraperitoneal challenge, Study was flawed due to low infection levels of *C. pecorum* in mice | [72] |
| *C. abortus* | 2004 | A - *C. abortus* inactivated in formaldehyde  B - *C. abortus* inactivated in β-propiolactone  C - *C. abortus* inactivated by binary ethylenimide  (2 doses for all) | A-Marcol 82 ne B-aluminium  Hydroxide  C1 - QS-21  C2 - Montanide ISA 773 | primiparous “Castellano” ewes | SC | The experimental vaccines induced considerably better protection than the two commercial ones. The new vaccine M7 especially showed no abortions, a good antibody response, the highest newborn lamb weights and the lowest level of C. abortus shedding at lambing. | 2 Commercial and 2 experimental vaccines. ID challenge to test abortion rates. | [73] |
| *C. trachomatis* (serovar D) | 2004 | *V. cholerae* ghosts with pKSEL5-2 plasmid containing MOMP or OMP1/2 gene inserts (3 doses) | *V. cholerae* ghosts | Female C57BL/6 mice | IM | Both vaccines induced a strong Th1 response to whole Chlamydia. OMP1/2 had a stronger response. | Immunological study and challenge trial to asses shedding | [74] |
| *C. pneumoniae* (K6) | 2004 | Recombinant Semliki Forest virus particles (rSFV) containing MOMP or Omp2 (3 doses) | Recombinant Semliki Forest virus particles (rSFV) | Female BALB/c mice | SC | Both MOMP and OMP2 induced partial protection post challenge. DNA priming per rSFV vaccine produced a hight INFγ post challenge at mucosal sites | Immunological study and intranasal challenge trial | [75] |
| *C. abortus* | 2004 | DNA vaccine for Heat shock protein (Hsp) GroEL (3 doses) | None | OF1 Mice | IM | No reduction in abortions or placental bacteria. Did induce IgG2a but this had no neutralizing effect | IP challenge on pregnant and non-pregant mice | [76] |
| *C. trachomatis* | 2004 | rPorB protein (2 doses) | Freund’s complete (1^st^ dose) & incomplete (2^nd^ dose) adjuvant | Swiss Webster mice | SC (1^st^ dose), IP (2^nd^ dose) | Weak Anti-PorB antibody response to vaccine alone but when already exposed to EBs this is increased to a strong response | IP challenge | [77] |
| *C. muridarum* | 2004 | MOMP | Cholera toxin | Male BALB/c mice | IN | High levels of MOMP-specific IgA in prostatic fluids (PF) and MOMP-specific IgA-secreting cells in the prostate. Up-regulation of PlgR in the prostate only. | Male study only on prostate IgA | [78] |
| *C. pneumoniae* (K6) | 2005 | DNA construct consisting of 8 genes:  Omp5, DnaK, Omp85, Cpn585, OmpB, PADRE, Cpn0562 and Cpn0928 (3 doses) | None | Female C57BL/6J and C57BL/6J-B2mtm1Unc mice. | IM and ID | Immunization of C57BL/6 mice primed IFNγ producing CD8+ CTL against all epitopes. CD8+ T cell lines secreted IFNγ and TNFα and exhibited CTL activity upon recognition of Cpn-infected macrophages and a 60-fold reduction at challenge. The MHC class 1 deficient mice indicated the responses were MCH class 1 dependent. | IN challenge | [79] |
| *C. trachomatis (MoPn)* | 2005 | Live EB’s | None | BALB/c mice | IN | Strong Chlamydia-specific humoral and cell-mediated immune responses were observed. A decrease in intensity and duration of shedding was observed post challenge (both routes) | IN or intravaginal challenge | [80] |
| *C. trachomatis (MoPn)* | 2005 | Native MOMP preparation | Montanide ISA 720 and CpG-1826 | BALB/c mice | IM and SC | 70% decrease vaginal shedding and 70% increase in pregnancies. Also developed strong Chlamydia-specific humoral and cellular immune responses | Ovarian bursa challenge then mated and pregnancies monitored | [81] |
| *C. trachomatis (MoPn)* | 2005 | Native MOMP preparation (3 doses) | MF59, LT-K63 or LT-R72 | BALB/c and C3H/HeN female mice | IM, SC and IN | Vaccination showed a non-significant decrease in shedding and increase in fertility. There were also small increases in serum and mucosal IgG and IgA but not-significant | left ovarian bursa challenge | [82] |
| *C. muridarum* | 2006 | Recombinant maltose binding protein-MOMP (4 doses) | cholera toxin and CpG oligodeoxynucleotide | Female BALB/c mice | IN or Transcutaneous (TC) | IN induced serum and mucosal IgG and IgA, TC induced serum and mucosal IgG only. IN resulted in twice as high INFγ than TC and 20-fold higher than control. Both vaccines lowered shedding IN <50 and TC <1500 IFU. Weight loss was only observed in non-vaccinated mice | IN challenge | [83] |
| *C. trachomatis (MoPn)* | 2006 | DNA - Codon altered MOMP and wild type MOMP | None | BALB/c mice | IM | Although the altered MOMP produced higher amounts of protein to the wild type there were no changes in IgG or mRNA expression between each Ag | In vitro challenge to compare antigens | [84, 85] |
| *C. trachomatis (MoPn)* | 2006 | Recombinant –MOMP with attached cholera toxin (2 doses) | cholera toxin | BALB/c mice | IN and intravaginal | Only IN showed increases in serum and mucosal IgA and IgG and a decrease in vaginal shedding post challenge | Intravaginal challenge | [86] |
| *C. pneumoniae* | 2006 | rMOMP and rHSP60 proteins or DNA (3 doses) | Pertussis toxin | CD8, Igh6 and IgA -/+ mice | IM or IP | DNA IN vaccine were protective against challenge. Only MOMP protein induced a mucosal response. IP showed no responses from any Ag. Also that IgA- mice were not protected | IN challenge | [87] |
| *C. psittaci (serovar D)* | 2006 | DNA MOMP in a eukaryotic expression vector | None | Turkeys | IM | The DNA vaccine persisted in turkey muscle for at >10 weeks and was expressed over the entire period. | Only studied the life span of the vaccine. | [88] |
| *C. muridarum* and *C. trachomatis* (serovar L2) | 2006 | Recombinant chlamydial protease-like activity factor (CPAF) from *C. trachomatis (serovar L2)* (3 doses) | IL-12 | C57BL/6 mice | IN | Vaccination induced antigen-specific CD4+ T cells that secreted elevated levels of IFNγ. This immunity could be transferred to naïve mice using CPAF-specific CD4+ T cells. Also found that mice deficient in MHCII lose their immunity. | Intravaginal challenge and the effect of CD4+ Tcells and Effects of CPAF on HLA-DR4 | [89, 90] |
| *C. muridarum* and *C. trachomatis* (serovar L2) | 2006 | Recombinant chlamydial protease-like activity factor (CPAF) from *C. trachomatis (serovar L2)* (3 doses) | IL-12 | BLAB/c mice | IN | Induced INFγ, serum IgG and IgA and mucosal IgG and IgA, decreased IL-4 compared to no adjuvant vaccination. There was a decrease in shedding and the development of pathology. | Intravaginal challenge | [91] |
| *C. pneumoniae (Kajaani 6)* | 2007 | Recombinant Heat-aggregated CopN protein (2 doses) | E. coli heat-labile toxin | BALB/c mice | IN | Immunization induced statistically significant protection against intranasal C. pneumoniae challenge with a strong immune response (INFγ, Ag-specific Abs and lymphocyte proliferation) | IN challenge | [92] |
| *C. trachomatis* (serovar D) and Herpes simplex virus type 2 | 2007 | Plasmid with MOMP and HSV2 glycoprotein D insert in Recombinant  *Vibrio cholerae* ghosts (rVCG) (3 doses) | Recombinant  *Vibrio cholerae* ghosts (rVCG) | female C57BL/6 mice | IM | Immunisation with the combination vaccine elicited serum and mucosal IgA and IgG2a antibodies to both chlamydial and HSV-2, and increased INFγ (in vitro) and showed protection to both targets post challenge. | Intravaginal challenge | [93] |
| *C. pneumoniae (strain AR39)* | 2007 | Recombinant  Omp-H, dnaK hsp70, HypoProt and LcrE proteins (3 doses each) | Freund’s complete (1^st^ dose) & incomplete (2^nd^ and 3^rd^ dose) adjuvant | Female BALB/c  mice | IP | Only LcrE induced CD4+ and CD8+ T cell activation, TH1 cytokine secretion and neutralising Abs and completely cleared challenge bacteria | IN challenge | [94] |
| *C. muridarum (Nigg II strain)* | 2007 | DNA for TC0512, TC0559, TC0757, TC0693, TC0462 and TC0767 in a eukaryotic expression vector (3 doses) | None | female BALB/c mice | intra-abdominally | TNF-α, IL-10, IL-4, IL-12 and IFN-γ were all detected in mice immunized. The most protective Ag was TC0512 (an outer membrane protein) increasing serum IgG and eliciting Th-1 and Th-2 cytokines. TC0512 also reduced shedding by 73%. Other Antigens also conferred protection but at lower levels than TC0512. | gene gun delivery of DNA and intravaginal challenge | [95] |
| *C. muridarum* and *C. trachomatis* (serovar L2) | 2007 | Recombinant chlamydial protease-like activity factor (CPAF) from *C. trachomatis (serovar L2)* (3 doses) | CpG deoxynucleotides | female BALB/c mice | IN or IP | Induced serum INFγ serum and mucosal IgA and displayed accelerated clearance and lowered symptoms compared to controls | Intravaginal challenge | [96] |
| *C. trachomatis* | 2007 | Hsp65-MOMP-T-epitopes (H-ctm1) and heat killed EBs (HK-EBS) (1 dose) | None | Female C57BL/6 mice | SC | Both H-ctm1 and HK-EBs accelerated clearance and lowered symptoms compared to controls. H-ctm1 was slightly more effective at clearance | Intravaginal challenge | [97] |
| *C. trachomatis* | 2007 | Plasmid with MOMP and or PorB insert in Recombinant  *Vibrio cholerae* ghosts (rVCG) (3 doses) | *Vibrio cholerae* ghosts | Female C57BL/6 mice | IM | Increased serum and mucosal IgA and IgG were detected. A combination vaccine induced a higher Th1 response than either single subunit vaccine. There was also accelerated clearance and lowered symptoms compared to controls. | Intravaginal challenge | [98] |
| *C. trachomatis* (serovar D) | 2007 | Live-attenuated Influenza A carrying 2 different immunodominant T-Cell epitopes from *C. trachomatis* MOMP (2 doses) | Live-attenuated Influenza A | Female C57BL/6 mice | IN | Resulted in a strong Th1 response upon challenge. Partial protection was also observed with increased clearance rate (P>0.002). Vaccination with multiple epitopes resulted in stronger Th1 response and mucosal IgA secretions. | Intravaginal challenge | [99] |
| *C. trachomatis* (serovar D and L2) | 2007 | rCPAF and/or rMOMP and/or rIncA from *C. trachomatis* (serovar D) | IL-12 | female BALB/c mice | IN | rCPAF gave the strongest IFNγ and serum antibody responses as a single antigen. Any multiple vaccination included with rCPAF gave increased clearance rates and decreased pathology. | Combination antigen verses single. Intravaginal challenge with *C. muridarum* | [100] |
| *C. muridarum* and *C. trachomatis* (serovar L2) | 2008 | rCPAF (3 doses) | CpG | female C57BL/6, BALB/c, C57BL/6 IFN-γ R-deficient  mice, BALB/c IFN-γ-deficient mice and C57BL/6 β_2_-microglobulin-deficient mice | IN | Early Ag-specific IFN- induction and CD4+ T cell infiltration correlated with increased clearance. IFNγ- competent CPAF-specific CD4+ T cells failed to enhance clearance within IFNγ-receptor-deficient mice. IFNγ production from adoptively transferred CPAF-specific CD4+ T cells was sufficient in IFNγ-deficient mice increase clearance and reduce pathology. | Intravaginal challenge to test the effect of IFNγ | [101] |
| *C. trachomatis* (serovar D) | 2008 | rMOMP or rNrdB (4 doses) | Cholera toxin and CpG oligodeoxynucleotide | female BALB/c mice | IN | rNrdB showed comparable CD4+ T-cell protection to that seen using whole EBs. Serum from rNrdB vaccinated mice also neutralized in-vitro Chlamydia cultures. | Intravaginal challenge | [102] |
| *C. muridarum (NiggII)* | 2008 | Native refolded MOMP (3 doses) | Th1-promoting cationic adjuvant formulation 1 (CAF01) or T helper cells type  2-promoting aluminum hydroxide (alum) | Female C57BL/6 mice | SC | Mice vaccinated with MOMP/alum had high IgG titers, low levels of IFN-y and TNF-α, and only a slight increase in clearance rates. Mice vaccinated with MOMP/CAF01 displayed high titers of IgG, IFN-y, and TNF-α and increased clearance rates. | Intravaginal challenge | [103] |
| *C. trachomatis* (serovar D) | 2008 | pORF5 DNA in a eukaryotic system (3 doses) | None | BALB/c mice | IN | Total and anti-pORF5 serum IgG and total mucosal IgA levels were elevated post vaccination. Th1 induced (measured by IFNγ). Decreased shedding and disease progression. | Intravaginal challenge with *C. muridarum* | [104] |
| *C. muridarum (NiggII)* | 2009 | DC transfected with PmpG-1 25-500, RplF, PmpE/F-2 25-575 or MOMP (3 doses) | None | Female C57BL/6 mice | IV | Reduced shedding at both challenge sites for Pmp/RplF vaccine, reduced shedding at genital site only for MOMP vaccine. PmpG worked the best on its own. | Pulmonary and cervicovaginal challenge | [105] |
| *C. trachomatis* (serovar K and E) | 2009 | Recombinant antigen encoded by CT694, CT695,  CT696, pmpG, CT089, CT858, and CT875 and UV inactive EBs (2 doses) | AS01B Ajuvant (MPL, QS21, and liposomes) | Female BALB/c and C57BL/6 mice | IM | All tested antigens showed some level of protection with the best being UV inactive EBs from serovar E. | Ectocervix or horn of the uterus challenge | [106] |
| *C. trachomatis* (serovar D) | 2009 | rVCG vaccine coexpressing chlamydial major outer membrane protein and CTA2B | Vibrio cholerae ghost | C57BL/6 mice | IM, Intravaginal and transcutaneous | Increased specific mucosal and systemic antibody and Th1 responses for both vaccine routes. | Intravaginal challenge with *C. muridarum* | [107] |
| *C. muridarum* | 2009 | MOMP prep in nanopartical vaults (3 doses) | nanopartical vaults | Female C57BL/6 mice | IN | Increased clearance, no activation of Toll like receptors decreased inflammation | Intravaginal challenge with *C. muridarum* | [108] |
| *C. muridarum (NiggII)* | 2009 | A recombinante protein of a putative type III secretion effector protein from Chlamydia (3 doses) | CpG emulsified in equal volume of incomplete Freund’s adjuvant | Female Balb/c mice | IM | Induced Th1-dominant immunity reduced shedding and the inflammatory pathologies in the fallopian tube tissues. | Intravaginal challenge with *C. muridarum* | [109] |
| *C. trachomatis* (serovar A) | 2009 | MOMP prep from serovar A (3 doses) | None | adult male cynomolgus macaques (Macaca fascicularis) | SC and IM | Immunization induced high serum IgG and IgA levels, with Abs displaying high strain-specific neutralizing activity. Decrease in infection levels but not clearance rates. | Ocular challenge | [110] |
| *C. muridarum* | 2009 | MOMP prep (4 doses) | CTA1-DD | Female BALB/c mice | IN | Reduced shedding and lead to neutralising systemic and mucosal antibodies. | Intravaginal challenge with *C. muridarum* | [111] |
| *C. trachomatis (MoPn)* | 2009 | Native and recombinant MOMP (nMOMP and rMOMP) | CpG-1826 and Montanide ISA 720 | BALB/c mice | IM and SC | Both nMOMP and rMOMP vaccinated mice lost less weight than the controls and showed lowered lung infections post challenge, with nMOMP outperforming rMOMP. | Intranasal challenge | [112] |
| *C. trachomatis* | 2009 | recombinant maltose binding MOMP fusion protein | CpG-ODN, Cholera toxin and/or Lipid C | female BALB/c mice | transcutaneous | All combinations of adjuvant gave similar cell-mediated and mucosal antibody immune responses with partial protection post challenge. | Adjuvant test trial with intravaginal and intranasal challenge | [113] |
| *C. trachomatis (MoPn)* | 2009 | Native MOMP prep | CpG oligodeoxynucleotide linked to the nontoxic B subunit of cholera toxin | BALB/c mice | IM and SC | High levels of serum Chlamydia-specific IgG antibodies and T-cell-mediated Chlamydia-specific immune responses. Also found IFN-γ-producing CD4 +, but not CD8 +, T-cells as having a significant correlation with challenge outcomes. | Intranasal challenge | [114] |
| *C. pecorum* | 2010 | 3 recombinant proteins MOMP, NrdB and TC0512 (Omp85) | Alhydrogel, Immunostimulating Complex (ISC) and TiterMax Gold | healthy female koalas | SC | All adjuvants induced strong serum neutralizing IgG and Chlamydia-specific PBMC proliferative responses. ISC also produced strong mucosal IgG response. | Adjuvant test trial | [115] |
| *C. pneumoniae* | 2010 | 18 different genes in plasmids contained in eukaryotic cells | Escherichia coli heat-labile enterotoxin gene inserted in a plasmid contained in eukaryotic cells | Inbred A/J female mice | Gene gun | Genes cutE and Cpn0420 conferred significant protection through the prevention of *C. pneumoniae*-induced death, reduction of lung disease and elimination of C. pneumoniae. Also Gene oppA 2 showed disease reduction *C. pneumoniae* elimination. | Antigenic screening trial with an intranasal challenge | [116] |
| *C. trachomatis* | 2010 | plasmid-free attenuated *Chlamydia trachomatis* L2-25667R (L2R) strain (1 dose) | None | Female C3H/HeJ mice | Intravaginal | Induced both chlamydial specific serum antibody and systemic CD4+ Th1 biased immune responses but failed to induce local IgA antibodies. Reduction in shedding | Intravaginal challenge of non-attenuated plasmid positive *C. trachomatis* (serovar D) | [117] |
| *C. muridarum* | 2010 | recombinant maltose binding protein (MBP)–MOMP fusion protein | cholera toxin (CT) and CpG oligodeoxynucleotides (CpG-ODN) | female BALB/c mice | Oral | 50% reduction in shedding, Increased production of IFNγ by splenic T cells and serum, MOMP specific IgG. Also detected low levels of mucosal, MOMP specific IgA. | Intravaginal challenge of *C. muridarum* | [118] |
| *C. muridarum* and *C. trachomatis* (serovar L2) | 2010 | recombinant chlamydial protease-like activity factor (rCPAF) active and heat inactivated | interleukin-12 | female BALB/c mice | intranasal | Active, but not inactive, rCPAF immunization induced high levels of anti-active CPAF antibody.  Both induced robust splenic CPAF-specific IFN- production and enhanced clearance. | Intravaginal challenge of *C. muridarum* | [119] |
| *C. muridarum* | 2010 | recombinant  proteins PmpG-1 and PmpE/F-2 and MOMP (3 doses) | Combinations of CpG Oligodeoxynucleotide, AbISCO-100, or DDA/TDB | Female C57BL/6 mice and BALB/c mice | SC | Vaccine - PmpG-1, PmpE/F-2 and MOMP with DDA/TDB had showed greatest protection. Adjuvant DDA/TDB had the highest IFNγ, TNFα and IL-17 responses. | Adjuvant test trial with intravaginal challenge of *C. muridarum* | [120] |
| *C. muridarum* | 2010 | recombinant maltose binding protein (MBP)–MOMP fusion protein (4 doses) | cholera toxin | male BALB/c mice | intranasal | Showed partial protective immunity, which significantly reduced the pathology. High titres of serum, MOMP-specific IgG1, IgG2a and IgA. | Intrapenile challenge. | [121] |
| *C. trachomatis* (serovar E) | 2010 | Transfected dendritic cells (DCs) with recombinant adenovirus carrying C. trachomatis serovar E major outer membrane protein gene | None | Mice | IV | Generated *C. trachomatis* specific Th1-biased cytokine production and mucosal IgA responses. Also lowered chlamydial loads and disease progression. | Intravaginal challenge | [122] |
| *C. felis* | 2010 | Formalin inactivated *C. felis* (2 doses) | ISA-70 | Male and female cats | SC | The new vaccine out produced the current commercial vaccine in all tests. There were significant increases in antibody production and decreased chlamydial loads with no adverse side effects. | Comparison of commercial vaccines to newly developed vaccine. Ocular and intranasal challenge | [123] |
| *C. muridarum* and *C. trachomatis* (serovar L2) | 2010 | rCPAF and UV-inactivated chlamydial  elementary bodies | recombinant CpG | female BALB/c mic | Intranasal | Increased serum, chlamydia specific antibodies, reduction in shedding with earlier clearance, reduced upper urogenital disease compared to single antigen and control vaccines | Intravaginal challenge of *C. muridarum* | [124] |
| *C. muridarum* (NiggII) and *C. trachomatis* (Serovar D) | 2010 | fusion protein composed of CT521 and CT443 (CTH1) (3 doses) | CAF01 | Female C3H/HeN and CB6F1 mice | SC | Reduced shedding, induced poly-functional T cells consisting of TNFα/IL-2 and TNFα/IL-2/IFN-γ positive cells. Also high titers of CTH1 specific IgG2a and IgG1. Lacks IgA response and complete protection | Compared intranasal infection to vaccine through Intravaginal challenge | [125] |
| *C. muridarum* | 2010 | Native MOMP | CpG-1826 and Montanide ISA 720 | Female C57BL/6 mice and B6.129S2-Igh-6^tm/Cgn^/J mice | SC | Reduced shedding with faster clearance. By depleting CD4+ or CD8+ T cells prior to vaccination they determined that protection was dependent on both CD4+ T cells and antibodies. | Intravaginal challenge of *C. muridarum* | [126] |
| *C. muridarum* | 2010 | Live *C. trachomatis* mouse pneumonitis biovar (MoPn) | None | female BALB/c mice | IN | Found no significant differences between immunized groups and controls. | Intranasal challenge of offspring from immunized mothers | [127] |
| *C. muridarum* (Nig II) | 2010 | Live *C. trachomatis* mouse pneumonitis biovar (MoPn) | None | Male BALB/c male | IN or intraurethral (i.u) | IU - 10%, IN – 28% and sham – 47% positive cultures from urethra organs. Increased clearance compared to controls for both IN and IU. Both IN and IU had increased IgG2a/IgG1 ratio and IFNγ. | Meatus urethra challenge | [128] |
| *C. muridarum* (Nig II) | 2010 | Recombinant MOMP | CpG and Montanide IM and SC. Cholera toxin, SL and CL | female BALB/c (H-2d) mice | IM, SC, Sublingual (SL) and colonic (CL) | High specific serum IgG titers and IgG2a/IgG1 ratios in SL + IM + SC and CL + IM. + SC.  SL + IM + SC group showed the best protection as shown by body weight. Combined routes also showed better clearance than mucosal only routes. | Vaccine route trial to test against intranasal challenge | [129] |
| *C. psittaci* | 2010 | live vector MOMP recombinant adenovirus (1 and 2 doses) | none | Male and female SPF chickens | Muscle or SC | All routes achieved a protective rate of 90% for a period of 6 months. | Testing vaccine dosage and route | [130] |
| *C. trachomatis* | 2010 | HPV type 6b capsid protein L1 and Ct MOMP multi-epitope chimeric DNA in a pcDNA3.1 vector | None | BALB/c mice | IM | Cytotoxicity was increased with HPV/MOMP as was IFN-7. No difference in IL4 or IL-10. | Test effects of a combined HPV and Chlamydia vaccine compared to just Chlamydia vaccine | [131] |
| *C. trachomatis* | 2011 | Recombinant PmpG or SctC | GNE | Pig | SC | PmpG showed increased protection but had very low antibody responses. SctC was ineffective. | Intravaginal challenge of *C. trachomatis* (serovar E) | [132] |
| *C. abortus* | 2011 | MOMP DNA in a pCI-neo plasmid (3 doses) | LBP3a | Female BALB/c mice | IM | Using the adjuvant increased serum IgG, T cell proliferation, IFNγ and IL-2 responses as well as chlamydia clearance. | Intraperitoneal challenge to compare adjuvant effect. | [133] |
| *C. muridarum* | 2011 | recombinant maltose binding protein (MBP)–MOMP fusion protein (4 doses) | Cholera toxin | Female BALB ⁄ c mice | IN | The administration of C. muridarum in conjunction with neutralizing antibodies reduced the numbers of mice infected, but this accelerated the development of pathology. | Vaginal lavage for the presence of neutralizing antibodies. These samples were then exposed to Chlamydia and administered to the vaginal vaults of mice | [134] |
| *C. trachomatis* (serovar E) | 2011 | Plasmid DNA (pWRG7079::MOMP) (2 doses) | GM-CSF, LTA and B and CpG | Pigs | intravaginal | showed significantly less macroscopic lesions, vaginal  excretion and chlamydial replication in the genital tract | Vaginal challenge GM-SCF given 1/52 prior vaccine | [135] |
| *C. trachomatis* | 2011 | Poring B and  polymorphic membrane protein-D proteins of *C. trachomatis* | Vibrio cholera ghosts | female C57BL/6  mice | IM | Increase in Chlamydia specific serum and mucosal IgG2a and IgA as well as splenic and genital T cell responses. Increased clearance. At 92 days post challenge a 2^nd^ challenge showed similar responses. | A double intravaginal challenge 92 days apart | [136] |
| *C. muridarum* | 2011 | Live and dead *C. muridarum* (heat or UV inactivated) (3 doses) | CpG (dead EB only) | Female C57BL/6 mice | Intranasal | Live vaccine (no adjuvant) outperformed all other vaccines with increased IFNγ and TNFα. Also found that live EBs pulsed with DCs presented 45 MHC class II mapping to 13 proteins compared to dead EBs with 6 MHC class II mapping to only 3 proteins (only 2 shared epitopes) | Intravaginal challenge | [137] |
| *C. trachomatis* | 2011 | Native MOMP | IC31 or Alum | BALB/c mice | IM and IC | IC31 increased serum, chlamydia specific IgG, splenic T-cells and IFNγ also less body weight loss compared to all other vaccines. | Intranasal challenge to test adjuvant effects | [138] |
| *C. muridarum* | 2011 | rCPAF (3 doses) | CpG | female BALB/c mice | Intranasal | rCPAF alone did not reduce infertility following primary challenge but did in 2^nd^ challenge. The addition of CpG increased the effectiveness to the primary challenge | Double intravaginal challenge to test fertility | [139] |
| *C. trachomatis* | 2011 | pcDNA3.1/HPV6bL1/CTMOMP (DNA plasmid) | None | Female BALB/c mice | IM | Increased antibodies against Ct MOMP, with Th1 and cytotoxic T lymphocyte activity against the Ct MOMP epitopes. Also cleared challenge infection 10 days faster than controls | Intravaginal challenge | [140] |
| *C. trachomatis* (serovar F) | 2011 | nMOMP (3 doses) | CpG-2395 and Montanide ISA 720 VG | rhesus macaques (Macaca mulatta) | IM and SC | Increased mucosal and serum Chlamydia specific IgG and IgA, serum neutralizing antibodies, IFNγ and TNFα | Immunogenicity trial | [141] |
| *C. trachomatis* | 2011 | Poring B and  polymorphic membrane protein-D proteins of *C. trachomatis* (3 doses) | Vibrio cholera ghosts | Female C57BL/6 mice | IM | Multi strain specific serum and mucosal antibody, Th1 and humoral immune responses were detected. Increased clearance was also noted. | Intravaginal challenge | [142] |
| *C. pneumoniae* | 2011 | rLcrE protein and plasmid (pδRC) DNA LcrE | None | BALB/c mice | IM | Using a DNA vaccine as a primer to protein vaccination significantly increased response and clearance rates | Intranasal challenge | [143] |
| *C. trachomatis* | 2011 | 32 different recombinant proteins in different combinations | LTK63/CpG | BALB/c mice | IM | Seven antigens showed partial protection when administered with LTK63/CpG adjuvant. Protection was largely the result of cellular immunity as assessed by CD4+ T-cell depletion. This was enhanced when combinations of the antigens were used. | Intranasal challenge with *C. muridarum* | [144] |
| *C. muridarum* | 2011 | nMOMP/Z3-14, nMOMP/A8-35,  rMOMP/Z3-14 or rMOMP/A8-35  (3 doses) | CpG and Montanide | female BALB/c mice | IM and SC | nMOMP outperformed rMOMP and nMOMP/A8-35 outperformed nMOMP/Z3-14. Performance was based on clearance and body and lung weight. | Differences in MOMP protein treatments (Zwitterionic detergent Z3-14 or amphipol A8-35).  Intranasal challenge | [145] |
| *C. muridarum* | 2011 | rMOMP | CpG and Montanide | BALB/c mice | Intravaginal, colonic, IN, sublingual, IM or SC | Strongest response was from a combination mucosal and systemic route. This increased clearance, and decreased pathology | Intravaginal challenge | [146] |
| *C. caviae* | 2011 | rMOMP | CpG-10109 and cholera  toxin | female guinea pigs | IN | High levels of mucosal and serum MOMP-specific IgG and IgA, with neutralising capabilities. Increase in clearance, and decrease in pathology. | Intravaginal challenge | [147] |
| *C. muridarum* | 2011 | rMOMP (3 doses) | Pam2CSK4, Poly (I:C), monophosphoryl lipid A, flagellin, imiquimod R837, imidazoquinoline R848, CpG-1826, M-Tri-DAP and muramyldipeptide. | Female BALB/c mice | IM | Ct-rMOMP + Pam2CSK4 showed a strong Th2 biased humoral immune response, with high Chlamydia-specific T cell proliferation and levels of IFNγ production. They also showed better protection to challenge. | Intranasal challenge | [148] |
| *C. muridarum* | 2011 | rMOMP (1 dose) | cholera toxin and CpG-ODN | Female BALB/c mice | Intranasal | Timing of vaccination affected the production of systemic antibodies and had minimal effects on mucosal antibodies. Vaccination during an active or resolved infection did not provide protection against re-exposure, and did not affect pathology outcomes. | Intravaginal challenge | [149] |
| *C. trachomatis* | 2011 | live-attenuated vaccine | None | Cynomolgus macaques | Ocular | Multiple infections with the attenuated plasmid-deficient strain produced no inflammatory ocular pathology but induced an anti-chlamydial immune response. Detected a difference in MHC class II alleles between partially and complete protection. | Ocular challenge with plasmid positive Ct strains. | [150] |
| *C. muridarum* | 2012 | recombinant adenovirus vector expressing the  antigen CPAF and rCPAF (2 dose) | CpG or HH2 | C3H/HeN and BALB/c mice | IN | AdCPAF stimulated potent antibody production but weak cellular immune responses.  The addition of a rCPAF CpG/HH2 induced a Th1 cellular response. An homologous prime/boost of rCPAF/CpG/HH2 induced a TH1/TH17 cellular response with poor antibody response. All treatments induced some level of protection. | IN and genital challenge. Main aim was to test prime/boost vaccination methods. | [151] |
| *C. muridarum* | 2012 | Live or UV inactivated EBs | CpG | Female Balb/c mice | IN or IM | IN with live organisms developed strong protection and a decrease in pathology related to increased IFNγ and antibodies but low IL-17.  IN with inactivated organisms or  IM with live or inactivated organisms produced high levels of IL-17 and still developed  UGT pathology. | Intravaginal challenge to compare IN Vs IM and Live Vs dead. | [152] |
| *C. pecorum* | 2012 | rMOMP and rNrdB (2 and 3 doses) | ISC | Koalas | SC | Strong specific, mucosal and serum antibody (including neutralizing antibodies) and lymphocyte proliferation responses were recorded in all vaccinated koalas | Vaccination of healthy and clinically diseased (conjunctivitis) | [153] |
| *C. muridarum* | 2012 | Recombinant proteins TC0075, TC0285, TC0419, GlgA, GlgB, GlgP and AGPAT (3 doses) | CpG, Incomplete Freunds Adjuvant | female Balb/c mice | IM | Only GlgP showed a protective response with increased specific antibodies and a Th1 cellular response with partial protection and a decreased pathology. | Intravaginal challenge | [154] |
| *C. muridarum* | 2012 | rPmpG (3 doses) | DDA-MPL, DDA-TDB, DDA-MMG, Montanide ISA720 –CpG-ODN1826, alum | Female C57BL/6 mice | SC | DDA-MPL and DDATDB  Showed best protective immune responses, multifunctional CD4+ T cells coexpressing IFNγ and TNFα, and reduced infection by more than 3 logs. | Adjuvant trial with a IN challenge | [155] |
| *C. muridarum* | 2012 | 13 different Recombinant proteins (3 doses) | DDA-MPL | Female C57BL/6 mice | SC | PmpG, PmpE, PmpF, Aasf, RplF, TC0420, and TC0825 gave results similar to rMOMP (positive control) | IN challenge | [155] |
| *C. trachomatis* | 2012 | MOMP-DNA in pWRG7079 plasmid | pcDNA3.1zeo::GM-CSF, PJV2004::LTa and PJV2005::LTb plasmids | Pigs | Intranasal, intravaginal or intradermal | Induced protection correlated with efficient T cell priming and significantly higher serum IgA titers. Infection could not be eradicated. Intradermal vaccination gave the lowest effective results. | Intravaginal challenge | [156] |
| *C. pneumoniae* | 2012 | CpnCTL7 or VR1012 plasmid DNA | None | female C57BL/6 mice | IM | CpnCTL7 conferred equal protection in the lungs of both aged and young mice. Was also partially effective in protecting against spread to the cardiovascular system of young mice, but failed to provide cardiovascular protection in aged animals. | IN challenge to see the effect of age on vaccine | [157] |
| *C. trachomatis* | 2012 | Recombinant proteins CT823 and CT144 (2 doses) | AbISCO-100 | Female C57BL/6 and BALB/c mice | SC | Stimulated CD8+ T cell responses, TH1 CD4+ T cell responses, and high protein-specific antibody responses. | Intravaginal challenge | [158] |
| *C. pecorum* | 2013 | rMOMP (3 doses) | ISC | Koalas | SC | Showed strong heterologous antibody and lymphocyte responses to MOMP protein. Also showed heterologous neutralizing antibodies to EBs | Trial to evaluate heterologous protection | [159] |
| *C. abortus* | 2013 | High dose live vaccine | None | Sheep | IN | Sheep administered high dose live EBs before pregnancy developed strong immune responses compared to low dose live EBs. Subsequent pregnancies resulted in a decrease in abortions of 100% in the high dose group compared to low dose. | Live vaccine before pregnancy to test effect on abortion after SC challenge during pregnancy | [160] |
| *C. muridarum* | 2013 | rArtJ, rOmcB, rMIP, rInc and rHP. (3 doses) | Incomplete Freunds Adjuvant | female Balb/c mice | IM | Only rMIP induced protection with specific antibody and Th1 responses resulting in decreased shedding and pathology. | Intravaginal challenge | [161] |
| *C. pneumoniae* | 2013 | rMOMP | CTA1-DD or cholera toxin/CpG | Mice (type not stated) | IN, sublingual (SL) and transcutaneous (TC) | SL and TC with MOMP and CT/CpG was the most protective, reducing chlamydial burden in the lungs and preventing weight loss. Also provided almost complete protection against fibrotic scarring in the lungs. This was due to antigen specific IFNγ, TNFα and IL-17. IN vaccine with 1 adjuvant only protected against infection but not pathology, the reverse was see for other routes. | Intranasal challenge | [162] |
| *C. muridarum* | 2013 | rMBP-MOMP and rNrdB (3 doses) | Cholera toxin and CpG-ODN | Female BALB/c mice | IN | Vaccine induced persistence in the oviducts. | Vaccination during infection. | [163] |
| *C. muridarum* | 2013 | rMOMP for Ct D, E and F and rMOMP from Cm (3 doses) | g CpG-1826 and Montanide  ISA 720 VG | Female BALB/c (H-2d) mice | IM and SC | Development of heterologous antibodies and cell mediated immune responses. Also all rMOMP vaccines reduced the number of recovered IFUs. | Trial to evaluate heterologous protection with IN challenge | [164] |
| *C. trachomatis* | 2013 | PLGA-rMOMP (nanoparticle encapsulated) | None | Female BALB/c mice | SC | Increased CD4+ and CD8+ T cells, rMOMP-specific IFNγ, IL-12p40 cytokines, serum specific immunoglobulin IgG and IgG2a. Notably, sera from PLGA-rMOMP immunized mice had a 64-fold higher Th1 than Th2 antibody titre. | Immunogenicity trial to test the effects of PLGA. | [165] |
| *C. pneumoniae* | 2013 | Live *C. pneumoniae* EBs | None | female C57BL/6 mice | IN | Increased Th1 response, clearance and a decrease in pathology compared to PBS vaccination. | Trial to asses cross protection from *C. pneu* vaccine to *C. muri* intravaginal challenge | [166] |
| *C. abortus* | 2013 | ompA-based lambda-phage-mediated DNA vaccine (3 dose) | None | Pigs | IM | Increased specific, serum IgG, lymphocytes, and total Tcell numbers. Also this response lasted for much longer than the live positive control. | Immunogenicity trial to test the effects of a phage-MOMP vaccine | [167] |
| *C. muridarum* | 2013 | rMBP-MOMP | Cholera toxin and CpG-ODN | Female BALB/c WT or IL-17 deficient mice | IN and Transcutaneous | Increased serum and mucosal, MOMP-specific IgG and IgA of IL-17-/- mice. Splenic T cell proliferation and IFNγ was greater in WT animals following  In-vitro re-stimulation, however vaccination was only effective at reducing infection in WT, not IL-17-/- mice. | Intravaginal challenge to see the effects of IL-17 on vaccine induced protection. | [168] |
| *C. pecorum* | 2013 | rMOMP (ompA G) | ISC | Koalas | SC | MOMP G vaccination showed heterologous identification of VD4. Natural infection showed homologous identification to VD1, 2 and 4. | Epitope mapping to establish level of cross protection compared to natural infection. | [169] |
| *C. muridarum* | 2013 | nMOMP with proteasome or Z3-14 (3 doses) | CpG þ Montanide | female BALB/c (H-2d) mice | IM and SC | Both nMOMPs developed strong humoral and cell mediated Chlamydia-specific Th1 immune responses. Both nMOMPs showed a similar level of protection based on body weight and lung IFUs. | IN challenge to test the effects of proteasome or Z3-14 treatment of nMOMP | [170] |
| *C. muridarum* | 2013 | rCPAF epitope fusion protein | CpG | HLA-DR4 (HLA-DRB1*0401) transgenic mouse | SC and IN | Identified at least 5 CPAF T cell epitopes presented by the HLA-DR4 complex. A fusion rCPAFep protein induced accelerated clearance and also showed cross protection to *C. trachomatis*. | Epitope screening trial and intranasal and intravaginal double challenge | [171] |
| *C. trachomatis* | 2014 | Multi-epitope fusion protein. (Human HLA-A2 and Mouse H-2-kd restricted CTLs, T helper cells and B cells) (3 doses) | Freund's adjuvant complete and incomplete | Balb/c mice | SC | High levels of specific antibodies (IgG1 and IgG2a) and IFNγ. Induced cytotoxic T cells. Increased clearance. | Intravaginal challenge | [172] |
| *C. muridarum* | 2014 | rMOMP (3 doses) | f Pam2CSK4 þ CpG-1826 and Montanide ISA 720 VG þ CpG-1826 | Female BALB/c mice | IN, colon. IM and SC | MOMP þ Pam2CSK4 þ CpG-1826 showed a strong Th2 response while MOMP þ Montanide ISA 720 VG þ CpG-1826 had a Th1 response (based on serum IgG). Both treatments increased clearance | IN challenge to test adjuvant combinations | [173] |
| *C. trachomatis* | 2014 | multi-epitope containing T- and B-cell  epitope-rich peptides on a hepatitis B surface antigen (3 doses) | None | Female Balb/c mice | IM | Specific antibodies and cytotoxic T cells to *Chlamydia* and HBsAg. Mucosal IgA and serum IgG increased to a max by week 8 and was significantly higher than control. Also vaccine combination  HB-*Chlamydia* was much more effective than *Chlamydia*-HB. | Immunogenicity and efficacy of the candidate vaccine and intravaginal challenge. | [174] |
| *C. trachomatis* | 2014 | M278 encapsulated in PLA-PEG (nanoparticle) (3 doses) | None | Female BALB/c mice | SC | Elicited higher M278-specific T-cell cytokines [Th1 (IFN-γ, IL-2), Th17 (IL-17)] and antibodies [Th1 (IgG2a), Th2 (IgG1, IgG2b)] compared to bare M278. | Test the immunogenicity of nanoparticle PLA-PEG with M278 | [175] |
| *C. pecorum* | 2014 | rMOMP (1 dose) | Tri-Adj (poly I:C, PCEP and IDR1002) | Koala | SC | Increase serum and mucosal IgG response and neutralizing antibodies 54/52 post vaccine. | Trial to show single dose effectiveness. | [176] |
| *C. muridarum* | 2014 | Recombinant ATP synthase complex TC0582 with/without TC0580, TC0581, TC0584 and MOMP. (3 doses) | CpG-1826 and Montanide ISA 720 VG | Female BALB/c mice | IM and SC | All combinations had a strong Chlamydia-specific humoral and cellular immune response. Based on weight and lung IFU counts, TC0582, TC0581 or MOMP showed significant protection. | IN challenge | [177] |
| *C. trachomatis* | 2014 | 14 different recombinant proteins. (3 doses) | CAF01 | C3H/HeN mice | SC | CT443, CT043, CT858, CT610, CT004 and CT681 antigens were found to be protective. Protection promoted by the two antigens CT043 and CT004 was mediated by CD4+ T-cells | Intravaginal challenge | [178] |
| *C. trachomatis* | 2014 | Plasmid deficient EBs (2yr post ocular vaccination) | None | Cynomolgus macaques | IM | Both solidly and partially protected macaques exhibited a CD4+ and CD8+ T cell response. CD8+ but not CD4+ T cells from solidly protected macaques proliferated against soluble chlamydial Ag. Depletion of CD8+ T cells in solidly protected macaques completely abrogated protective immunity | Boost vaccinated 2yrs after initial ocular dose | [179] |
| *C. muridarum* | 2014 | nMOMP solubilized with either A8-35 or Z3-14 (4 doses) | CpG-1826 and Montanide ISA 720 VG | female BALB/c mice | Colonic (1^st^ and 2^nd^ dose), IM and SC (3^rd^ and 4^th^ dose) | A8-35 had a higher ratio of Abs to denatured EBs over live EB, recognized more synthetic MOMP peptides, had higher neutralizing and IFNγ titres than sera from mice immunized with Z3-14. A8-35 also showed better clearance rates. | Intravaginal challenge | [180] |
| *C. muridarum* | 2014 | Recombinant proteins PmpE, PmpF, PmpG, PmpH and MOMP in different combinations (3 doses) | DDA/MPL | C57BL/6, Balb/c and C3H mice | SC | PmpEFGH + MOMP gave the best response. Pmps elicited more variable cellular immune responses than MOMP among the three strains of mice. PmpEFGH + MOMP accelerated clearance. | Intravaginal challenge | [181] |
| *C. muridarum* | 2014 | recombinant pGP3 or pGP4 (3 doses) | Aluminium hydroxide Gel | C57BL/6N mice | SC | lower chlamydial burden in the lungs, lower IFN- level | IN challenge | [182] |
| *C. abortus* | 2015 | rPmp18D (3 doses) | CpG1826 + FL or Vibrio  cholera ghosts | Female C57BL/6 mice | IN | rVCG-Pmp18D-immunized mice elicited more robust antigen-specific IFNγ, IgA and IgG2c responses as well as an improved clearance. | Intravaginal challenge | [183] |
| *C. trachomatis* | 2015 | Recombinant proteins extVD4E, extVD4F, Hirep1, Hirep2, CTH522 and rMOMP (VD4 proteins) (3 doses) | CAF01 | Female B6C3F1 and C3H/HeN mic | SC and IN | Produced a strong immune response (IgG only) that was cross protective and increased clearance. No IgA increase. | Intravaginal challenge | [184] |
| *C. trachomatis* | 2015 | Multi-epitope of major outer  membrane protein (MOMPm) With Hepatitis B virus core antigen (HBcAg) (3 doses) | None | Female BALB/c mice | SC | IgG, IgA, cytokines and T-cell levels all showed an improved response when the MOMPm was coupled with HBcAg (as compared to MOMPm alone). The MOMPm-HBcAG also showed increased protection compared to MOMPm alone. | Intravaginal challenge | [185] |
| *C. pecorum* | 2015 | rMOMP | ISC | Male Koalas | SC and IN | SC elicited stronger cell-mediated responses in PBMCs and greater plasma antibody levels whereas the IN elicited stronger mucosal (UGT) humoral responses. | Trial to test vaccination route | [186] |
| *C. trachomatis* | 2015 | Recombinant proteins Hirep1 (prime and boost) and CTH93 (prime only) (2 doses) | CAF01 | Göttingen Minipig | IM (prime) and IN (Boost) | Priming induced IFNγ and IL-17A with high neutralizing IgG. Boosting (post challenge) elicited a strong mucosal IgA response and increased clearance. | Trial to test the effect of a prime–infection–boost treatment protocol. | [187] |
| *C. psittaci* | 2015 | rHVT-pmpD-N (HVT – herpesvirus of turkeys) | None | SPF chickens | SC | There was no decrease in the HVT clearance. Also elicited a decrease in respiratory pathology, lesions and *Chlamydia* load. | Trial to test the effect of combining a commercial vaccine for HVT and *C. psittaci*) throat challenge (*C. psittaci)* | [188] |
| *C. trachomatis* | 2015 | UV-inactive EBs | charge-switching synthetic adjuvant particles (cSAPs) | C57BL/6 and BALB/c mice | Intrauterine, SC or IN | Elicited long-lived protection  in conventional and humanized mice. Vaccine without cSAPs were found in tolerogenic T cells whereas vaccine with cSAPs were found in immunogenic uterine DCs. Only mucosal route led to Trm cells which lead to increased clearance. | Intrauterine challenge | [189] |
| *C. trachomatis* | 2015 | rMOMP, rPmpD, *pgp3-*GST and MOMP in Adenovirus, modified vaccinia Ankara (MVA) (3 doses) | MF59 – IM  monophosphoryl Lipid A - IN | Female BALB/c mice | IM and IN | rMOMP showed an increase of anti-MOMP ocular antibodies. IM gave a higher Mucosal IgG response. All other proteins gave leaker responses. | Antibody response study. No challenge. | [190] |
| *C. muridarum* | 2015 | nMOMP (1 dose) | Montanide+CpG or  Alum+CpG | C3H/HeN female mice | IM and SC | MOMP+Montanide+CpG developed high levels of C. muridarum-specific antibodies, with a high IgG2a/IgG1 ratio and neutralizing titres and gave 80% protection. Alum+CpG had low antibody levels and failed to give any protection | ovarian bursa challenge to test pregnancy rates | [191] |
| *C. trachomatis* | 2015 | PmpC in probiotic  Escherichia coli Nissle 1917 bacterial ghosts (EcN BGs) (3 doses) | None | BALB/c mice and Guinea pigs | SC or conjunctiva | NPmpC-specific mucosal IgA levels in tears were significantly increased when immunized via the conjunctiva in mice. Both methods showed increased IFNγ. Conjunctiva vaccine resulted in lowered pathology in guinea pigs. | Antibody trial in mice with an ocular challenge trial in guinea pigs. | [192] |
| *C. pecorum* | 2016 | rMOMP (3 doses) | ISC | Koala | SC | There was not a significant drop in *Chlamydia* infection but there was a significant decrease in disease which was stronger at 6 months than at 12 months. | Trial to test the long term activity of the vaccine in health koalas | [193] |
| *C. trachomatis* | 2016 | fusion proteins  (Hirep1 and CTH93) (2 doses) | CAF01 | Göttingen minipig | IM | Induced a strong CMI response against the vaccine antigens and high titers of antibodies, particularly against the VD4 region of MOMP. There was also an increase in protection. | Intravaginal challenge | [194] |
| *C. pecorum* | 2016 | rMOMP (3 doses) | ISC | Koala | SC | Produced a unique set of specific epitope-directed antibodies that were responsible for in vitro neutralisation activity. | Trial to compare antibody profiles between vaccinated koalas and un-vaccinated healthy and diseased koalas | [195] |
| *C. trachomatis* | 2016 | MOMP transgene inserted into Human Adenovirus 5 or modified vaccinia Ankara vectors( 2, 3, 4 and 5 doses | MF59 | Female BALB/c and B6C3F1 mice | IM | DNA-HuAd5-MVA Protein vaccine induced a CMI response with a Th1-biased serum antibody response and high serum and vaginal MOMP-specific antibodies and enhanced clearance. | Intravaginal challenge | [196] |
| *C. pecorum* | 2016 | rMOMP (1 dose – TriAdj and 3 dose –ISC) | ISC or TriAdj (poly I:C, PCEP and IDR-1002) | Koala | SC | Overall, both adjuvants produced strong Chlamydia-specific cellular (IFN-γ and IL-17A) responses in circulating PBMCs as well as MOMP-specific and functional, in vitro neutralising antibodies | Adjuvant effect trial | [197] |
| *C. psittaci* | 2016 | rCPSIT_p8(3 doses) | Complete and incomplete Freund’s adjuvant | BALB/c mice | IP | Decreased load in the lungs, lowered IFN-γ, and reduced the extent of inflammation. CPSIT_p8-specific antibodies did not have a neutralizing capacity. | Intranasal challenge | [198] |
| *C. trachomatis* | 2016 | rCopB, rCopD and rCT584 fusion protein (2 doses) | CpG ODN1826 | female C57Bl/6 mice | IN | Elicited serum neutralizing antibodies that inhibited *C. trachomatis* infection in vitro. 95% reduction in shedding and 87.5% in pathology (*C. mur*) | Intravaginal challenge with *C. muridarum*. | [199] |
| *C. trachomatis* | 2016 | rPmpD (3 doses) | second-generation lipid adjuvant (SLA) | female C57BL/6 mice | SC | Significantly enhanced resistance to infection and reduction in mean bacterial load. Also found that anti-rPmpD antibodies recognize Ct EBs. Increase in mucosal IgG. | Intravaginal challenge | [200] |
| *C. trachomatis* | 2016 | rMOMP (3 doses) | SPA08-1 to SPA08-4 | CD1 mice | IM | Increase in phosphate substitution decreased the adsorptive coefficient and adsorptive capacity for both rMOMP and E6020.  Phosphate substitution also decrease in the % adsorption of  Ser E rMOMP.  An increase in phosphate substitution of the SPA08 adjuvant resulted in an increase in specific serum IgG and IgG1. | Trial to test the effects of phosphate substitution of the adjuvant | [201] |
| *C. trachomatis* | 2016 | rN-PmpC | *Lactobacillus rhamnosus* (LB) | BALB/c mice | Eye drops | With LB the vaccine initiated a T cell response with an elevated percentage of CD25+ T cells and CD8+ effector T cells, enhanced CD4+ Th1 | Tested the effects of the antigen with and without the adjuvant with | [202] |
| *C. pecorum* | 2016 | rMOMP | TriAdj (poly I:C, PCEP and IDR-1002) | Koala | SC | Reduced/eliminated current infection and reduced pathology | Tested the effects of a vaccine on current ocular disease | [203] |
| *C. trachomatis* | 2016 | rMOMP (based on VS2/4) (2 doses) | cholera toxin (intravaginal) | C57BL/6 mice | IN | Reduced shedding, local and systemic IgG1, IgG2c and IgA responses and IFNγ, IL13 and IL17 and decreased pathology | Intravaginal challenge | [204] |
| *C. trachomatis* | 2017 | MOMP multi-epitope and HPV16L2 genes carried in pcDNA plasmid (4 doses) | None | BALB/c mice | IM | Induced higher levels of anti-Ct IgG, anti-HPV16L2 IgG in serum and IgA at the mucosa. Increased protection and decreased pathology | Intravaginal challenge | [205] |
| *C. muridarum* | 2017 | rTC_0037 (type III secretion system protein) | monophosphoryl lipid A | Mice | IN and SC | Induced T-cell responses, increased protection and reduced pathology. | Intravaginal challenge | [206] |
| *C. psittaci* | 2017 | rCPSIT_0846 (3 doses) | Complete and incomplete Freund’s adjuvant | BALB/c mice | IP | Developed strong T-lymphocyte responses displaying strong Th1 cytokines (IFNγ) and humoral immune response with increased serum specific IgG. Reduced shedding and pathology. | Intranasal challenge | [207] |
| *C. muridarum* | 2017 | rPmpG packaged into vault nanoparticles (3 doses) | None | female C57 BL mice | IN | Significantly reduced shedding and pathology with multiple cytokine responses. | Intravaginal challenge | [208] |
| *C. muridarum* | 2017 | rMOMP and nMOMP | CAF01 and CAF09 | BALB/c mice | SC and IN | All four groups of mice immunized with CAF01, or CAF09 and MOMP had an increase in specific antibodies and increased IFNγ and IL17 and decreased levels of IL4. Reduced shedding and pathology was also observed. nMOMP out performed rMOMP | Intranasal challenge | [209] |
| *C. trachomatis* | 2017 | Hirep2 (H2), anchored to the surface of *L. plantarum* cells (1 prime and 2 boost doses) | CAF01 | Female B6C3F1 mice | SC (prime), IN (boost) | Increased cellular responses and mucosal specific IgA. | Prime (antigen only) boost (antigen on *L. plantarum)* vaccine (no challenge) | [210] |
| *C. abortus* | 2017 | Attenuated live EBs | None | Sheep | SC | Results suggest that negative affective state induced by chronic stress treatment may induce a stronger inflammatory response to vaccine challenge in sheep. | Test the effects of stress on vaccine effectiveness. | [211] |
| *C. trachomatis* | 2017 | rCPAF | CpG | Guinea pigs | IN | Significantly reduced shedding and pathology. Increased serum specific IgG and IFNγ | Intravaginal challenge | [212] |
| *C. trachomatis* | 2017 | Recombinant fragments from all 9 Pmps and MOMP (3 doses) | CpG-1826 and Montanide ISA 720 | BALB/c mice | IM and SC | Pmp C, G or H showed the highest protection. Although none out performed MOMP | Intranasal challenge using *C. muridarum* | [213] |
| *C. muridarum* | 2017 | rPmpA (2 doses) | CpG-ODN 1826 | C57Bl/6 mice | SC | Decreased shedding and pathology. Adjuvant only increased pathology. | Intravaginal challenge | [214] |
| *C. trachomatis* | 2017 | rCTH522 or Hirep1 with CTH93 | CAF01 | Female B6C3F1 mice | SC and/or IN | IFNγ, IL17 and serum specific IgG were increased by all vaccines. But only duel route vaccination elicited a Mucosal specific IgA response. Although no improvement in shedding or pathology was seen between routes. | Intravaginal challenge to test the difference in vaccination route | [215] |
| *C. trachomatis* | 2017 | MOMP-HBcAg with 3 added epitopes (not named) (3 doses) | None | BALB/c female mice | SC | Improved immunogenicity as seen in IFNγ, IgG, IgA and clearance. A combination of all 3 outperformed all other combinations. | Intravaginal challenge | [216] |
| *C. pecorum* | 2017 | rMOMP or rPmp (1 dose) | TriAdj (Poly I:C, PCEP and IDR-1002) | Koalas | SC | Both antigens elicited an increased specific serum and mucosal IgG and mucosal IgA, Increased IFNγ and IL17. Only rMOMP showed protection from infection. | Trial to test the effects of different antigens. | [217] |
| *C. trachomatis* | 2017 | PorB or PmpD contained in Vibrio cholera ghosts (3 doses) | Vibrio cholera ghosts | female C57BL/6 mice | Rectal | Elicited high levels of mucosal and serum chlamydial-specific IFNγ and humoral immune responses. Showed cross protection among two CT genotypes and reduced shedding and pathology. | Intravaginal challenge | [218] |
| *C. pecorum* | 2017 | rMOMP and rPmpG (1 and 2 doses) | TriAdj (Poly I:C, PCEP and IDR-1002) | Lambs and ewes. | SC | SC-vaccination response was poor for ewes. SC-vaccination in lambs increased serum and mucosal anti-MOMP-G and anti-PmpG IgG and IgA. 2 doses caused increases in the height and duration humoral responses. Antigen-specific IFNγ was produced in the PBMCs. | Vaccination of pregnant ewes and lambs to test the immune response in the different aged sheep. | [219] |
| *C. muridarum* | 2017 | rMOMP, rPmpG, T3SS, CdcF, TCo873 IncA and TC0500. (4 doses) | ISCOMATRIX, TriAdj (Poly I:C, PCEP and IDR-1002), CTA1-DD and ADVAX | Female mice (BALB/c) | SC, IN | Comparison of single and double antigen vaccines largely targeting the extracellular stage, elicited significant yet comparable protection against vaginal shedding. Using adjuvants and different routes further enhanced protection. Greatest protection was from antigens that targeted multiple stages of the chlamydia life cycle. | Intravaginal challenge to test various different combinations of antigens and adjuvants | [220] |
| *C. trachomatis* | 2017 | Dendrimers (G4OH), bound to (Pep4, AFPQFRSATLLL) through an ester bond (3 doses) | None | Female BALB/c mice | SC | Increased protection, reduced infections load and pathology. | Test the effects of binding a peptide to a dendrimer for vaccination. Intravaginal challenge. | [221] |

1. Morgan, H.R. and R.W. Wiseman, *Growth of Psittacosis Virus in Roller Tube Tissue Culture; Use in a Vaccine.* The Journal of Infectious Diseases, 1946. **79**(2): p. 131-133.

2. Sampaio, A.A., et al., *STUDIES ON TRACHOMA. IV. INVESTIGATIONS IN PORTUGAL ON FORMALIN-KILLED TRACHOMA VACCINES WITH SPECIAL REFERENCE TO SEROLOGIC RESPONSE.* The American journal of tropical medicine and hygiene, 1963. **12**: p. 909-915.

3. Chang, H.L., H.Y. Chin, and K.C. Wang, *EXPERIMENTAL STUDIES ON TRACHOMA VACCINE IN MONKEYS.* Chinese medical journal, 1964. **83**: p. 755-762.

4. Mitzel, J.R., G.G. Wright, and N.S. Swack, *Cross Immunity Among Strains of Chlamydia psittaci.* Proceedings of the Society for Experimental Biology and Medicine, 1970. **135**(3): p. 944-946.

5. McKercher, D.G., et al., *Experimentally induced immunity to chlamydial abortion of cattle.* Journal of Infectious Diseases, 1973. **128**(2): p. 231-234.

6. Nichols, R.L., E.S. Murray, and P.E. Nisson, *Use of enteric vaccines in protection against chlamydial infections of the genital tract and the eye of guinea pigs.* Journal of Infectious Diseases, 1978. **138**(6): p. 742-746.

7. Senyk, G., et al., *Cell-mediated immune responses to chlamydial antigens in guinea pigs injected with inactivated chlamydiae.* Medical Microbiology and Immunology, 1980. **168**(2): p. 91-101.

8. Malaty, R., et al., *Serum and tear antibodies to Chlamydia after reinfection with guinea pig inclusion conjunctivitis agent.* Investigative Ophthalmology and Visual Science, 1981. **21**(6): p. 833-841.

9. MacDonald, A.B., D. McComb, and L. Howard, *Immune response of owl monkeys to topical vaccination with irradiated Chlamydia trachomatis.* Journal of Infectious Diseases, 1984. **149**(3): p. 439-442.

10. Johnson, F.W.A. and D. Hobson, *Intracerebral infection of mice with ovine strains of Chlamydia psittaci: An animal screening test for the assay of vaccines.* Journal of Comparative Pathology, 1986. **96**(5): p. 497-505.

11. Whittum-Hudson, J.A., R.A. Prendergast, and H.R. Taylor, *Changes in conjunctival lymphocyte populations induced by oral immunization with chlamydia trachomatis.* Current Eye Research, 1986. **5**(12): p. 973-979.

12. Rodolakis, A. and A. Souriau, *Response of goats to vaccination with temperature-sensitive mutants of Chlamydia psittaci obtained by nitrosoguanidine mutagenesis.* American Journal of Veterinary Research, 1986. **47**(12): p. 2627-2631.

13. Taylor, H.R. and R.A. Prendergast, *Attempted oral immunization with chlamydial lipopylsaccharide subunit vaccine.* Investigative Ophthalmology and Visual Science, 1987. **28**(10): p. 1722-1726.

14. Taylor, H.R., et al., *Oral immunization against chlamydial eye infection.* Investigative Ophthalmology and Visual Science, 1987. **28**(2): p. 249-258.

15. Rekiki, A., A. Bouakane, and A. Rodolakis, *Combined vaccination of live 1B Chlamydophila abortus and killed phase I Coxiella burnetii vaccine does not destroy protection against chlamydiosis in a mouse model.* Canadian Journal of Veterinary Research, 2004. **68**(3): p. 226-228.

16. Wills, J.M., et al., *Effect of vaccination on feline Chlamydia psittaci infection.* Infection and Immunity, 1987. **55**(11): p. 2653-2657.

17. Taylor, H.R., et al., *Oral immunization with chlamydial major outer membrane protein (MOMP).* Investigative Ophthalmology and Visual Science, 1988. **29**(12): p. 1847-1853.

18. Cui, Z.D., et al., *Immunoprophylaxis of Chlamydia trachomatis lymphogranuloma venereum pneumonitis in mice by oral immunization.* Infection and Immunity, 1989. **57**(3): p. 739-744.

19. Rank, R.G., B.E. Batteiger, and L.S.F. Soderberg, *Immunization against chlamydial genital infection in guinea pigs with UV-inactivated and viable chlamydiae administered by different routes.* Infection and Immunity, 1990. **58**(8): p. 2599-2605.

20. Tan, T.W., et al., *Protection of sheep against Chlamydia psittaci infection with a subcellular vaccine containing the major outer membrane protein.* Infection and Immunity, 1990. **58**(9): p. 3101-3108.

21. Anderson, I.E., et al., *Efficacy against ovine enzootic abortion of an experimental vaccine containing purified elementary bodies of Chlamydia psittaci.* Veterinary Microbiology, 1990. **24**(1): p. 21-27.

22. Wilsmore, A.J., et al., *Clinical and immunological responses of ewes following vaccination with an experimental formalin-inactivated Chlamydia psittaci (ovis) vaccine and subsequent challenge with the live organism during pregnancy.* British Veterinary Journal, 1990. **146**(4): p. 341-348.

23. Wilsmore, A.J., et al., *Protection of ewes vaccinated with A22 strain Chlamydia psittaci (ovis) against challenge in pregnancy with homologous and heterologous strains of the organism.* British Veterinary Journal, 1990. **146**(4): p. 349-353.

24. Hansen, D.E., et al., *Efficacy of a vaccine to prevent Chlamydia- or Campylobacter-induced abortions in ewes.* Journal of the American Veterinary Medical Association, 1990. **196**(5): p. 731-734.

25. Tuffrey, M., et al., *Heterotypic protection of mice against chlamydial salpingitis and colonization of the lower genital tract with a human serovar F isolate of Chlamydia trachomatis by prior immunization with recombinant serovar L1 major outer-membrane protein.* Journal of General Microbiology, 1992. **138**(8): p. 1707-1715.

26. Cheng, X., et al., *Characterization of the humoral response induced by a peptide corresponding to variable domain IV of the major outer membrane protein of Chlamydia trachomatis serovar E.* Infection and Immunity, 1992. **60**(8): p. 3428-3432.

27. Davidson, H.J., S.A. Byrnes, and P.C. Montgomery, *The effect of immunization route on rat serum and tear antibody responses to Chlamydia trachomatis.* Regional Immunology, 1993. **5**(2): p. 114-119.

28. Su, H. and H.D. Caldwell, *Immunogenicity of a synthetic oligopeptide corresponding to antigenically common T-helper and B-cell neutralizing epitopes of the major outer membrane protein of Chlamydia trachomatis.* Vaccine, 1993. **11**(11): p. 1159-1166.

29. Murdin, A.D., et al., *A poliovirus hybrid expressing a neutralization epitope from the major outer membrane protein of Chlamydia trachomatis is highly immunogenic.* Infection and Immunity, 1993. **61**(10): p. 4406-4414.

30. Qu, Z., et al., *Analysis of the humoral response elicited in mice by a chimeric peptide representing variable segments I and IV of the major outer membrane protein of Chlamydia trachomatis.* Vaccine, 1994. **12**(6): p. 557-564.

31. Pal, S., et al., *Protection against infertility in a BALB/c mouse salpingitis model by intranasal immunization with the mouse pneumonitis biovar of Chlamydia trachomatis.* Infection and Immunity, 1994. **62**(8): p. 3354-3362.

32. Gajdosová, E., et al., *The immunogenicity of a vaccine against enzootic abortion in sheep.* Veterinarni Medicina, 1994. **39**(10): p. 589-596.

33. Westbay, T.D., et al., *Dissociation of immune determinants of outer membrane proteins of Chlamydia psittaci strain guinea pig inclusion conjunctivitis.* Infection and Immunity, 1994. **62**(12): p. 5614-5623.

34. Su, H., M. Parnell, and H.D. Caldwell, *Protective efficacy of a parenterally administered MOMP-derived synthetic oligopeptide vaccine in a murine model of Chlamydia trachomatis genital tract infection: serum neutralizing IgG antibodies do not protect against chlamydial genital tract infection.* Vaccine, 1995. **13**(11): p. 1023-1032.

35. Knight, S.C., et al., *A peptide of Chlamydia trachomatis shown to be a primary T-cell epitope in vitro induces cell-mediated immunity in vivo.* Immunology, 1995. **85**(1): p. 8-15.

36. Campos, M., et al., *A chlamydial major outer membrane protein extract as a trachoma vaccine candidate.* Investigative Ophthalmology and Visual Science, 1995. **36**(8): p. 1477-1491.

37. Jones, G.E., et al., *Efficacy trials with tissue-culture grown, inactivated vaccines against chlamydial abortion in sheep.* Vaccine, 1995. **13**(8): p. 715-723.

38. Sandbulte, J., et al., *Evaluation of Chlamydia psittaci subfraction and subunit preparations for their protective capacities.* Veterinary Microbiology, 1996. **48**(3-4): p. 269-282.

39. Peterson, E.M., et al., *The effect of orientation within a chimeric peptide on the immunogenicity of Chlamydia trachomatis epitopes.* Molecular Immunology, 1996. **33**(4-5): p. 335-339.

40. Whittum-Hudson, J.A., et al., *Oral immunization with an anti-idiotypic antibody to the exoglycolipid antigen protects against experimental Chlamydia trachomatis infection.* Nature Medicine, 1996. **2**(10): p. 1116-1121.

41. Pal, S., et al., *Immunization with an acellular vaccine consisting of the outer membrane complex of Chlamydia trachomatis induces protection against a genital challenge.* Infection and Immunity, 1997. **65**(8): p. 3361-3369.

42. Chalmers, W.S.K., et al., *Use of a live chlamydial vaccine to prevent ovine enzootic abortion.* Veterinary Record, 1997. **141**(3): p. 63-67.

43. Zhang, D.J., et al., *DNA vaccination with the major outer-membrane protein gene induces acquired immunity to Chlamydia trachomatis (mouse pneumonitis) infection.* Journal of Infectious Diseases, 1997. **176**(4): p. 1035-1040.

44. Su, H., et al., *Vaccination against chlamydial genital tract infection after immunization with dendritic cells pulsed ex vivo with nonviable Chlamydiae.* Journal of Experimental Medicine, 1998. **188**(5): p. 809-818.

45. Pal, S., et al., *Vaccination of mice with DNA plasmids coding for the Chlamydia trachomatis major outer membrane protein elicits an immune response but fails to protect against a genital challenge.* Vaccine, 1999. **17**(5): p. 459-465.

46. Zhang, D.J., et al., *Characterization of immune responses following intramuscular DNA immunization with the MOMP gene of Chlamydia trachomatis mouse pneumonitis strain.* Immunology, 1999. **96**(2): p. 314-321.

47. Zhang, D., et al., *Immunity to Chlamydia trachomatis mouse pneumonitis induced by vaccination with live organisms correlates with early granulocyte-macrophage colony-stimulating factor and interleukin-12 production and with dendritic cell-like maturation.* Infection and Immunity, 1999. **67**(4): p. 1606-1613.

48. Motin, V.L., L.M. De La Maza, and E.M. Peterson, *Immunization with a peptide corresponding to chlamydial heat shock protein 60 increases the humoral immune response in c3h mice to a peptide representing variable domain 4 of the major outer membrane protein of chlamydia trachomatis.* Clinical and Diagnostic Laboratory Immunology, 1999. **6**(3): p. 356-363.

49. Vanrompay, D., et al., *Protection of turkeys against Chlamydia psittaci challenge by gene gun- based DNA immunizations.* Vaccine, 1999. **17**(20-21): p. 2628-2635.

50. Peterson, E.M., et al., *Intranasal immunization with Chlamydia trachomatis, serovar E, protects from a subsequent vaginal challenge with the homologous serovar.* Vaccine, 1999. **17**(22): p. 2901-2907.

51. Vanrompay, D., et al., *Turkeys are protected from infection with Chlamydia psittaci by plasmid DNA vaccination against the major outer membrane protein.* Clinical and Experimental Immunology, 1999. **118**(1): p. 49-55.

52. Pal, S., et al., *Immunogenic and protective ability of the two developmental forms of Chlamydiae in a mouse model of infertility.* Vaccine, 1999. **18**(7-8): p. 752-761.

53. Brunham, R.C. and D.J. Zhang, *Transgene as vaccine for chlamydia.* American Heart Journal, 1999. **138**(5 II): p. S519-S522.

54. Svanholm, C., et al., *Protective DNA immunization against Chlamydia pneumoniae.* Scandinavian Journal of Immunology, 2000. **51**(4): p. 345-353.

55. Zhang, D.J., et al., *Priming with Chlamydia trachomatis major outer membrane protein (MOMP) DNA followed by MOMP ISCOM boosting enhances protection and is associated with increased immunoglobulin A and Th1 cellular immune responses.* Infection and Immunity, 2000. **68**(6): p. 3074-3078.

56. Murdin, A.D., et al., *Use of a mouse lung challenge model to identify antigens protective against Chlamydia pneumoniae lung infection.* Journal of Infectious Diseases, 2000. **181**(6 SUPPL. 3): p. S544-S551.

57. Igietseme, J.U. and A. Murdin, *Induction of protective immunity against Chlamydia trachomatis genital infection by a vaccine based on major outer membrane protein-lipophilic immune response-stimulating complexes.* Infection and Immunity, 2000. **68**(12): p. 6798-6806.

58. Penttilä, T., et al., *Immunity to Chlamydia pneumoniae induced by vaccination with DNA vectors expressing a cytoplasmic protein (Hsp60) or outer membrane proteins (MOMP and Omp2).* Vaccine, 2000. **19**(9-10): p. 1256-1265.

59. Whittum-Hudson, J.A., et al., *The anti-idiotypic antibody to chlamydial glycolipid exoantigen (GLXA) protects mice against genital infection with a human biovar of Chlamydia trachomatis.* Vaccine, 2001. **19**(28-29): p. 4061-4071.

60. Pal, S., et al., *Immunization with the Chlamydia trachomatis mouse pneumonitis major outer membrane protein can elicit a protective immune response against a genital challenge.* Infection and Immunity, 2001. **69**(10): p. 6240-6247.

61. Shaw, J., et al., *Dendritic cells pulsed with a recombinant chlamydial major outer membrane protein antigen elicit a CD4+ type 2 rather than type 1 immune response that is not protective.* Infection and Immunity, 2002. **70**(3): p. 1097-1105.

62. Pal, S., et al., *Immunization with the Chlamydia trachomatis mouse pneumonitis major outer membrane protein by use of CpG oligodeoxynucleotides as an adjuvant induces a protective immune response against an intranasal chlamydial challenge.* Infection and Immunity, 2002. **70**(9): p. 4812-4817.

63. Lu, H., Z. Xing, and R.C. Brunham, *GM-CSF transgene-based adjuvant allows the establishment of protective mucosal immunity following vaccination with inactivated Chlamydia trachomatis.* Journal of Immunology, 2002. **169**(11): p. 6324-6331.

64. Bandholtz, L., et al., *Adjuvant modulation of the immune responses and the outcome of infection with Chlamydia pneumoniae.* Clinical and Experimental Immunology, 2002. **130**(3): p. 393-403.

65. Donati, M., et al., *DNA immunization with pgp3 gene of Chlamydia trachomatis inhibits the spread of chlamydial infection from the lower to the upper genital tract in C3H/HeN mice.* Vaccine, 2003. **21**(11-12): p. 1089-1093.

66. Pal, S., et al., *Immunization with the Chlamydia trachomatis major outer membrane protein, using the outer surface protein A of Borrelia burgdorferi as an adjuvant, can induce protection against a chlamydial genital challenge.* Vaccine, 2003. **21**(13-14): p. 1455-1465.

67. Eko, F.O., et al., *Recombinant Vibrio cholerae ghosts as a delivery vehicle for vaccinating against Chlamydia trachomatis.* Vaccine, 2003. **21**(15): p. 1694-1703.

68. Knitz, J.C., et al., *Humoral immune response in sows vaccinated with a bacterin prepared from a herd-derived Chlamydophila abortus strain.* Deutsche Tierarztliche Wochenschrift, 2003. **110**(9): p. 369-374.

69. Pal, S., E.M. Peterson, and L.M. De La Maza, *Induction of protective immunity against a Chlamydia trachomatis genital infection in three genetically distinct strains of mice.* Immunology, 2003. **110**(3): p. 368-375.

70. Caro, M.a.R., et al., *Relationship between the immune response and protection conferred by new designed inactivated vaccines against ovine enzootic abortion in a mouse model.* Vaccine, 2003. **21**(23): p. 3126-3136.

71. Berry, L.J., et al., *Transcutaneous Immunization with Combined Cholera Toxin and CpG Adjuvant Protects against Chlamydia muridarum Genital Tract Infection.* Infection and Immunity, 2004. **72**(2): p. 1019-1028.

72. Rekiki, A., et al., *Efficacy of live Chlamydophila abortus vaccine 1B in protecting mice placentas and foetuses against strains of Chlamydophila pecorum isolated from cases of abortion.* Veterinary Microbiology, 2004. **99**(3-4): p. 295-299.

73. García De La Fuente, J.N., et al., *Efficacy of different commercial and new inactivated vaccines against ovine enzootic abortion.* Veterinary Microbiology, 2004. **100**(1-2): p. 65-76.

74. Eko, F.O., et al., *A novel recombinant multisubunit vaccine against Chlamydia.* Journal of Immunology, 2004. **173**(5): p. 3375-3382.

75. Penttilä, T., et al., *DNA immunization followed by a viral vector booster in a Chlamydia pneumoniae mouse model.* Vaccine, 2004. **22**(25-26): p. 3386-3394.

76. Héchard, C., O. Grépinet, and A. Rodolakis, *Molecular cloning of the Chlamydophila abortus groEL gene and evaluation of its protective efficacy in a murine model by genetic vaccination.* Journal of Medical Microbiology, 2004. **53**(9): p. 861-868.

77. Kawa, D.E., J. Schachter, and R.S. Stephens, *Immune response to the Chlamydia trachomatis outer membrane protein PorB.* Vaccine, 2004. **22**(31-32): p. 4282-4286.

78. Hickey, D.K., et al., *Intranasal immunization with C. muridarum major outer membrane protein (MOMP) and cholera toxin elicits local production of neutralising IgA in the prostate.* Vaccine, 2004. **22**(31-32): p. 4306-4315.

79. Pinchuk, I., et al., *A CD8+ T cell heptaepitope minigene vaccine induces protective immunity against Chlamydia pneumomiae.* Journal of Immunology, 2005. **174**(9): p. 5729-5739.

80. Pal, S., E.M. Peterson, and L.M. De La Maza, *Vaccination of newborn mice induces a strong protective immune response against respiratory and genital challenges with Chlamydia trachomatis.* Vaccine, 2005. **23**(46-47): p. 5351-5358.

81. Pal, S., E.M. Peterson, and L.M. De La Maza, *Vaccination with the chlamydia trachomatis major outer membrane protein can elicit an immune response as protective as that resulting from inoculation with live bacteria.* Infection and Immunity, 2005. **73**(12): p. 8153-8160.

82. Pal, S., et al., *Immunization with the Chlamydia trachomatis major outer membrane protein, using adjuvants developed for human vaccines, can induce partial protection in a mouse model against a genital challenge.* Vaccine, 2006. **24**(6): p. 766-775.

83. Skelding, K.A., et al., *Comparison of intranasal and transcutaneous immunization for induction of protective immunity against Chlamydia muridarum respiratory tract infection.* Vaccine, 2006. **24**(3): p. 355-366.

84. Zheng, Y., et al., *Codon modification of the Chlamydia trachomatis MOMP gene enhances the immune responses in DNA-vaccinated mice.* Chinese Journal of Microbiology and Immunology, 2006. **26**(1): p. 92-96.

85. Zheng, Y., et al., *Codon usage bias in Chlamydia trachomatis and the effect of codon modification in the MOMP gene on immune responses to vaccination.* Biochemistry and Cell Biology, 2007. **85**(2): p. 218-226.

86. Singh, S.R., et al., *Mucosal immunization with recombinant MOMP genetically linked with modified cholera toxin confers protection against Chlamydia trachomatis infection.* Vaccine, 2006. **24**(8): p. 1213-1224.

87. Rodríguez, A., et al., *Immunoglobulin A and CD8+ T-cell mucosal immune defenses protect against intranasal infection with Chlamydia pneumoniae.* Scandinavian Journal of Immunology, 2006. **63**(3): p. 177-183.

88. Loots, K., et al., *Evaluation of the persistence and gene expression of an anti-Chlamydophila psittaci DNA vaccine in turkey muscle.* BMC Veterinary Research, 2006. **2**.

89. Murphey, C., et al., *The protective efficacy of chlamydial protease-like activity factor vaccination is dependent upon CD4+ T cells.* Cellular Immunology, 2006. **242**(2): p. 110-117.

90. Murthy, A.K., et al., *Chlamydial protease-like activity factor induces protective immunity against genital chlamydial infection in transgenic mice that express the human HLA-DR4 allele.* Infection and Immunity, 2006. **74**(12): p. 6722-6729.

91. Murthy, A.K., et al., *Intranasal vaccination with a secreted chlamydial protein enhances resolution of genital Chlamydia muridarum infection, protects against oviduct pathology, and is highly dependent upon endogenous gamma interferon production.* Infection and Immunity, 2007. **75**(2): p. 666-676.

92. Tammiruusu, A., et al., *Intranasal administration of chlamydial outer protein N (CopN) induces protection against pulmonary Chlamydia pneumoniae infection in a mouse model.* Vaccine, 2007. **25**(2): p. 283-290.

93. Macmillan, L., et al., *A recombinant multivalent combination vaccine protects against Chlamydia and genital herpes.* FEMS Immunology and Medical Microbiology, 2007. **49**(1): p. 46-55.

94. Thorpe, C., et al., *Discovery of a vaccine antigen that protects mice from Chlamydia pneumoniae infection.* Vaccine, 2007. **25**(12): p. 2252-2260.

95. McNeilly, C.L., et al., *Expression library immunization confers partial protection against Chlamydia muridarum genital infection.* Vaccine, 2007. **25**(14): p. 2643-2655.

96. Cong, Y., et al., *Intranasal immunization with chlamydial protease-like activity factor and CpG deoxynucleotides enhances protective immunity against genital Chlamydia muridarum infection.* Vaccine, 2007. **25**(19): p. 3773-3780.

97. Yang, S.R., et al., *Protective effects of fusion protein of Hsp65-MOMP-T-epitopes on C. trachomatis genital tract infection of mice.* Journal of Jilin University Medicine Edition, 2007. **33**(3): p. 440-444.

98. Ifere, G.O., et al., *Immunogenicity and protection against genital Chlamydia infection and its complications by a multisubunit candidate vaccine.* Journal of Microbiology, Immunology and Infection, 2007. **40**(3): p. 188-200.

99. He, Q., et al., *Live-attenuated influenza viruses as delivery vectors for Chlamydia vaccines.* Immunology, 2007. **122**(1): p. 28-37.

100. Li, W., et al., *Induction of cross-serovar protection against genital chlamydial infection by a targeted multisubunit vaccination approach.* Clinical and Vaccine Immunology, 2007. **14**(12): p. 1537-1544.

101. Li, W., et al., *Antigen-specific CD4+ T cells produce sufficient IFN-γ to mediate robust protective immunity against genital Chlamydia muridarum infection.* Journal of Immunology, 2008. **180**(5): p. 3375-3382.

102. Barker, C.J., et al., *In silico identification and in vivo analysis of a novel T-cell antigen from Chlamydia, NrdB.* Vaccine, 2008. **26**(10): p. 1285-1296.

103. Hansen, J., et al., *Liposome delivery of Chlamydia muridarum major outer membrane protein primes a Th1 response that protects against genital chlamydial infection in a mouse model.* Journal of Infectious Diseases, 2008. **198**(5): p. 758-767.

104. Li, Z., et al., *Immunization with chlamydial plasmid protein pORF5 DNA vaccine induces protective immunity against genital chlamydial infection in mice.* Science in China, Series C: Life Sciences, 2008. **51**(11): p. 973-980.

105. Yu, H., et al., *Novel Chlamydia muridarum T cell antigens induce protective immunity against lung and genital tract infection in murine models.* Journal of Immunology, 2009. **182**(3): p. 1602-1608.

106. Coler, R.N., et al., *Identification and characterization of novel recombinant vaccine antigens for immunization against genital Chlamydia trachomatis.* FEMS Immunology and Medical Microbiology, 2009. **55**(2): p. 258-270.

107. Ekong, E.E., et al., *A Vibrio cholerae ghost-based subunit vaccine induces cross-protective chlamydial immunity that is enhanced by CTA2B, the nontoxic derivative of cholera toxin.* FEMS Immunology and Medical Microbiology, 2009. **55**(2): p. 280-291.

108. Champion, C.I., et al., *A vault nanoparticle vaccine induces protective mucosal immunity.* PLoS ONE, 2009. **4**(4).

109. Wang, J., et al., *A chlamydial type III-secreted effector protein (Tarp) is predominantly recognized by antibodies from humans infected with Chlamydia trachomatis and induces protective immunity against upper genital tract pathologies in mice.* Vaccine, 2009. **27**(22): p. 2967-2980.

110. Kari, L., et al., *Chlamydia trachomatis native major outer membrane protein induces partial protection in nonhuman primates: Implication for a trachoma transmission- blocking vaccine.* Journal of Immunology, 2009. **182**(12): p. 8063-8070.

111. Cunningham, K.A., et al., *CTA1-DD is an effective adjuvant for targeting anti-chlamydial immunity to the murine genital mucosa.* Journal of Reproductive Immunology, 2009. **81**(1): p. 34-38.

112. Sun, G., et al., *Protection against an intranasal challenge by vaccines formulated with native and recombinant preparations of the Chlamydia trachomatis major outer membrane protein.* Vaccine, 2009. **27**(36): p. 5020-5025.

113. Hickey, D.K., F.E. Aldwell, and K.W. Beagley, *Transcutaneous immunization with a novel lipid-based adjuvant protects against Chlamydia genital and respiratory infections.* Vaccine, 2009. **27**(44): p. 6217-6225.

114. Cheng, C., et al., *Induction of protective immunity by vaccination against Chlamydia trachomatis using the major outer membrane protein adjuvanted with CpG oligodeoxynucleotide coupled to the nontoxic B subunit of cholera toxin.* Vaccine, 2009. **27**(44): p. 6239-6246.

115. Carey, A.J., et al., *A Multi-subunit chlamydial vaccine induces antibody and cell-mediated immunity in immunized koalas (phascolarctos cinereus): Comparison of three different adjuvants.* American Journal of Reproductive Immunology, 2010. **63**(2): p. 161-172.

116. Li, Y., et al., *Novel Chlamydia pneumoniae vaccine candidates confirmed by Th1-enhanced genetic immunization.* Vaccine, 2010. **28**(6): p. 1598-1605.

117. Olivares-Zavaleta, N., et al., *Immunization with the attenuated plasmidless Chlamydia trachomatis L2(25667R) strain provides partial protection in a murine model of female genitourinary tract infection.* Vaccine, 2010. **28**(6): p. 1454-1462.

118. Hickey, D.K., F.E. Aldwell, and K.W. Beagley, *Oral immunization with a novel lipid-based adjuvant protects against genital Chlamydia infection.* Vaccine, 2010. **28**(7): p. 1668-1672.

119. Chaganty, B.K.R., et al., *Heat denatured enzymatically inactive recombinant chlamydial protease-like activity factor induces robust protective immunity against genital chlamydial challenge.* Vaccine, 2010. **28**(11): p. 2323-2329.

120. Yu, H., et al., *Chlamydia muridarum T-cell antigens formulated with the adjuvant DDA/TDB induce immunity against infection that correlates with a high frequency of gamma interferon (IFN-γ)/tumor necrosis factor alpha and IFN-γ/ interleukin-17 double-positive CD4+ T cells.* Infection and Immunity, 2010. **78**(5): p. 2272-2282.

121. Cunningham, K.A., et al., *CD4+ T cells reduce the tissue burden of Chlamydia muridarum in male BALB/c mice.* Vaccine, 2010. **28**(31): p. 4861-4863.

122. Lü, H., et al., *Dendritic cells (DCs) transfected with a recombinant adenovirus carrying chlamydial major outer membrane protein antigen elicit protective immune responses against genital tract challenge infection.* Biochemistry and Cell Biology, 2010. **88**(4): p. 757-765.

123. Masubuchi, K., et al., *Efficacy of a new inactivated Chlamydophila felis vaccine in experimentally-infected cats.* Journal of Feline Medicine and Surgery, 2010. **12**(8): p. 609-613.

124. Li, W., et al., *Immunization with a combination of integral chlamydial antigens and a defined secreted protein induces robust immunity against genital chlamydial challenge.* Infection and Immunity, 2010. **78**(9): p. 3942-3949.

125. Olsen, A.W., et al., *Protection against chlamydia promoted by a subunit vaccine (CTH1) compared with a primary intranasal infection in a mouse genital challenge model.* PLoS ONE, 2010. **5**(5).

126. Farris, C.M., S.G. Morrison, and R.P. Morrison, *CD4+ T cells and antibody are required for optimal major outer membrane protein vaccine-induced immunity to Chlamydia muridarum genital infection.* Infection and Immunity, 2010. **78**(10): p. 4374-4383.

127. Pal, S., O. Tatarenkova, and L.M. de la Maza, *Maternal immunity partially protects newborn mice against a Chlamydia trachomatis intranasal challenge.* Journal of Reproductive Immunology, 2010. **86**(2): p. 151-157.

128. Pal, S., A.K. Sarcon, and L.M. de la Maza, *A new murine model for testing vaccines against genital Chlamydia trachomatis infections in males.* Vaccine, 2010. **28**(48): p. 7606-7612.

129. Ralli-Jain, P., et al., *Enhancement of the protective efficacy of a Chlamydia trachomatis recombinant vaccine by combining systemic and mucosal routes for immunization.* Vaccine, 2010. **28**(48): p. 7659-7666.

130. Qiu, C., et al., *Immunization trials with an avian chlamydial MOMP gene recombinant adenovirus.* Bioengineered Bugs, 2010. **1**(4): p. 267-273.

131. Shi, Z.H., et al., *The cellular immune response produced in BALB/c mice immunized with HPV6b L1/Ct MOMP multi-epitope chimeric DNA.* Chinese Journal of Microbiology and Immunology, 2010. **30**(10): p. 942-948.

132. Schautteet, K., et al., *Validation of the Chlamydia trachomatis genital challenge pig model for testing recombinant protein vaccines.* Journal of Medical Microbiology, 2011. **60**(1): p. 117-127.

133. Ling, Y., et al., *Co-administration of the polysaccharide of lycium barbarum with DNA vaccine of chlamydophila abortus augments protection.* Immunological Investigations, 2011. **40**(1): p. 1-13.

134. Cunningham, K.A., et al., *Chlamydia muridarum Major Outer Membrane Protein-Specific Antibodies Inhibit In Vitro Infection but Enhance Pathology In Vivo.* American Journal of Reproductive Immunology, 2011. **65**(2): p. 118-126.

135. Schautteet, K., et al., *Protection of pigs against Chlamydia trachomatis challenge by administration of a MOMP-based DNA vaccine in the vaginal mucosa.* Vaccine, 2011. **29**(7): p. 1399-1407.

136. Eko, F.O., et al., *Induction of immune memory by a multisubunit chlamydial vaccine.* Vaccine, 2011. **29**(7): p. 1472-1480.

137. Yu, H., et al., *Immunization with live and dead chlamydia muridarum induces different levels of protective immunity in a murine genital tract model: Correlation with MHC class II peptide presentation and multifunctional Th1 cells.* Journal of Immunology, 2011. **186**(6): p. 3615-3621.

138. Cheng, C., et al., *Induction of protection in mice against a respiratory challenge by a vaccine formulated with the Chlamydia major outer membrane protein adjuvanted with IC31®.* Vaccine, 2011. **29**(13): p. 2437-2443.

139. Murthy, A.K., et al., *Vaccination with the defined chlamydial secreted protein CPAF induces robust protection against female infertility following repeated genital chlamydial challenge.* Vaccine, 2011. **29**(14): p. 2519-2522.

140. Xu, W., et al., *Protective immunity against Chlamydia trachomatis genital infection induced by a vaccine based on the major outer membrane multi-epitope human papillomavirus major capsid protein L1.* Vaccine, 2011. **29**(15): p. 2672-2678.

141. Cheng, C., et al., *Immunogenicity of a vaccine formulated with the Chlamydia trachomatis serovar F, native major outer membrane protein in a nonhuman primate model.* Vaccine, 2011. **29**(18): p. 3456-3464.

142. Eko, F.O., et al., *Evaluation of a broadly protective Chlamydia-cholera combination vaccine candidate.* Vaccine, 2011. **29**(21): p. 3802-3810.

143. Faludi, I. and A. Míra Szabó, *Vaccination with dna vector expressing chlamydial low calcium response protein e (lcre) against chlamydophila pneumoniae infection.* Acta Microbiologica et Immunologica Hungarica, 2011. **58**(2): p. 123-134.

144. Finco, O., et al., *Approach to discover T- and B-cell antigens of intracellular pathogens applied to the design of Chlamydia trachomatis vaccines.* Proceedings of the National Academy of Sciences of the United States of America, 2011. **108**(24): p. 9969-9974.

145. Tifrea, D.F., et al., *Amphipols stabilize the Chlamydia major outer membrane protein and enhance its protective ability as a vaccine.* Vaccine, 2011. **29**(28): p. 4623-4631.

146. Carmichael, J.R., et al., *Induction of protection against vaginal shedding and infertility by a recombinant Chlamydia vaccine.* Vaccine, 2011. **29**(32): p. 5276-5283.

147. Andrew, D.W., et al., *Partial protection against chlamydial reproductive tract infection by a recombinant major outer membrane protein/CpG/cholera toxin intranasal vaccine in the guinea pig Chlamydia caviae model.* Journal of Reproductive Immunology, 2011. **91**(1-2): p. 9-16.

148. Cheng, C., et al., *A TLR2 agonist is a more effective adjuvant for a chlamydia major outer membrane protein vaccine than ligands to other TLR and NOD receptors.* Vaccine, 2011. **29**(38): p. 6641-6649.

149. Carey, A., et al., *A comparison of the effects of a chlamydial vaccine administered during or after a C. muridarum urogenital infection of female mice.* Vaccine, 2011. **29**(38): p. 6505-6513.

150. Kari, L., et al., *A live-attenuated chlamydial vaccine protects against trachoma in nonhuman primates.* Journal of Experimental Medicine, 2011. **208**(11): p. 2217-2223.

151. Brown, T.H.T., et al., *Comparison of immune responses and protective efficacy of intranasal prime-boost immunization regimens using adenovirus-based and CpG/HH2 adjuvanted-subunit vaccines against genital Chlamydia muridarum infection.* Vaccine, 2012. **30**(2): p. 350-360.

152. Lu, C., et al., *Protective immunity against mouse upper genital tract pathology correlates with high IFNγ but low IL-17 T cell and anti-secretion protein antibody responses induced by replicating chlamydial organisms in the airway.* Vaccine, 2012. **30**(2): p. 475-485.

153. Kollipara, A., et al., *Vaccination of healthy and diseased koalas (Phascolarctos cinereus) with a Chlamydia pecorum multi-subunit vaccine: Evaluation of immunity and pathology.* Vaccine, 2012. **30**(10): p. 1875-1885.

154. Li, Z., et al., *Induction of protective immunity against chlamydia muridarum intravaginal infection with a chlamydial glycogen phosphorylase.* PLoS ONE, 2012. **7**(3).

155. Yu, H., et al., *Chlamydia muridarum T cell antigens and adjuvants that induce protective immunity in mice.* Infection and Immunity, 2012. **80**(4): p. 1510-1518.

156. Schautteet, K., et al., *Protection of pigs against genital Chlamydia trachomatis challenge by parenteral or mucosal DNA immunization.* Vaccine, 2012. **30**(18): p. 2869-2881.

157. Eddens, T., et al., *Effect of age and vaccination on extent and spread of Chlamydia pneumoniae infection in C57BL/6 mice.* Immunity and Ageing, 2012. **9**.

158. Picard, M.D., et al., *High-throughput proteomic screening identifies Chlamydia trachomatis antigens that are capable of eliciting T cell and antibody responses that provide protection against vaginal challenge.* Vaccine, 2012. **30**(29): p. 4387-4393.

159. Kollipara, A., et al., *Antigenic specificity of a monovalent versus polyvalent MOMP based Chlamydia pecorum vaccine in koalas (Phascolarctos cinereus).* Vaccine, 2013. **31**(8): p. 1217-1223.

160. Longbottom, D., et al., *Intranasal Infection with Chlamydia abortus Induces Dose-Dependent Latency and Abortion in Sheep.* PLoS ONE, 2013. **8**(2).

161. Lu, C., et al., *Induction of protective immunity against Chlamydia muridarum intravaginal infection with the chlamydial immunodominant antigen macrophage infectivity potentiator.* Microbes and Infection, 2013. **15**(4): p. 329-338.

162. O'Meara, C.P., et al., *Immunization with a MOMP-Based Vaccine Pfrotects Mice against a Pulmonary Chlamydia Challenge and Identifies a Disconnection between Infection and Pathology.* PLoS ONE, 2013. **8**(4).

163. Carey, A.J., et al., *Characterization of in vitro chlamydia muridarum persistence and utilization in an in vivo mouse model of chlamydia vaccine.* American Journal of Reproductive Immunology, 2013. **69**(5): p. 475-485.

164. Tifrea, D.F., et al., *Vaccination with the recombinant major outer membrane protein elicits antibodies to the constant domains and induces cross- serovar protection against intranasal challenge with chlamydia trachomatis.* Infection and Immunity, 2013. **81**(5): p. 1741-1750.

165. Fairley, S.J., et al., *Chlamydia trachomatis recombinant MOMP encapsulated in PLGA nanoparticles triggers primarily T helper 1 cellular and antibody immune responses in mice: A desirable candidate nanovaccine.* International Journal of Nanomedicine, 2013. **8**: p. 2085-2099.

166. Manam, S., et al., *Intranasal Vaccination with Chlamydia pneumoniae Induces Cross-Species Immunity against Genital Chlamydia muridarum Challenge in Mice.* PLoS ONE, 2013. **8**(5).

167. Ou, C., et al., *Evaluation of an ompA-based phage-mediated DNA vaccine against Chlamydia abortus in piglets.* International Immunopharmacology, 2013. **16**(4): p. 505-510.

168. Andrew, D.W., et al., *The Duration of Chlamydia muridarum Genital Tract Infection and Associated Chronic Pathological Changes Are Reduced in IL-17 Knockout Mice but Protection Is Not Increased Further by Immunization.* PLoS ONE, 2013. **8**(9).

169. Kollipara, A., et al., *Vaccination of Koalas with a Recombinant Chlamydia pecorum Major Outer Membrane Protein Induces Antibodies of Different Specificity Compared to Those Following a Natural Live Infection.* PLoS ONE, 2013. **8**(9).

170. Tifrea, D.F., et al., *Vaccination with major outer membrane protein proteosomes elicits protection in mice against a Chlamydia respiratory challenge.* Microbes and Infection, 2013. **15**(13): p. 920-927.

171. Li, W., et al., *A T cell epitope-based vaccine protects against chlamydial infection in HLA-DR4 transgenic mice.* Vaccine, 2013. **31**(48): p. 5722-5728.

172. Tu, J., et al., *A multi-epitope vaccine based on Chlamydia trachomatis major outer membrane protein induces specific immunity in mice.* Acta Biochimica et Biophysica Sinica, 2014. **46**(5): p. 401-408.

173. Cheng, C., et al., *A vaccine formulated with a combination of TLR-2 and TLR-9 adjuvants and the recombinant major outer membrane protein elicits a robust immune response and significant protection against a Chlamydia muridarum challenge.* Microbes and Infection, 2014. **16**(3): p. 244-252.

174. Zhu, S., et al., *Hepatitis B virus surface antigen as delivery vector can enhance Chlamydia trachomatis MOMP multi-epitope immune response in mice.* Applied Microbiology and Biotechnology, 2014. **98**(9): p. 4107-4117.

175. Dixit, S., et al., *Poly(lactic acid)-poly(ethylene glycol) nanoparticles provide sustained delivery of a Chlamydia trachomatis recombinant MOMP peptide and potentiate systemic adaptive immune responses in mice.* Nanomedicine: Nanotechnology, Biology, and Medicine, 2014. **10**(6): p. 1311-1321.

176. Khan, S.A., et al., *Vaccination of koalas (Phascolarctos cinereus) with a recombinant chlamydial major outer membrane protein adjuvanted with poly I: C, a host defense peptide and polyphosphazine, elicits strong and long lasting cellular and humoral immune responses.* Vaccine, 2014. **32**(44): p. 5781-5786.

177. Cheng, C., et al., *Assessment of the role in protection and pathogenesis of the Chlamydia muridarum V-type ATP synthase subunit A (AtpA) (TC0582).* Microbes and Infection, 2014. **16**(2): p. 123-133.

178. Olsen, A.W., P. Andersen, and F. Follmann, *Characterization of protective immune responses promoted by human antigen targets in a urogenital Chlamydia trachomatis mouse model.* Vaccine, 2014. **32**(6): p. 685-692.

179. Olivares-Zavaleta, N., et al., *CD8+ T cells define an unexpected role in live-attenuated vaccine protective immunity against chlamydia trachomatis infection in macaques.* Journal of Immunology, 2014. **192**(10): p. 4648-4654.

180. Tifrea, D.F., et al., *Increased immunoaccessibility of MOMP epitopes in a vaccine formulated with amphipols may account for the very robust protection elicited against a vaginal challenge with chlamydia muridarum.* Journal of Immunology, 2014. **192**(11): p. 5201-5213.

181. Yu, H., et al., *Evaluation of a multisubunit recombinant polymorphic membrane protein and major outer membrane protein T cell vaccine against Chlamydia muridarum genital infection in three strains of mice.* Vaccine, 2014. **32**(36): p. 4672-4680.

182. Mosolygó, T., et al., *Protection promoted by pGP3 or pGP4 against Chlamydia muridarum is mediated by CD4+ cells in C57BL/6N mice.* Vaccine, 2014. **32**(40): p. 5228-5233.

183. Pan, Q., et al., *Comparative evaluation of the protective efficacy of two formulations of a recombinant Chlamydia abortus subunit candidate vaccine in a mouse model.* Vaccine, 2015. **33**(15): p. 1865-1872.

184. Olsen, A.W., et al., *Protection Against Chlamydia trachomatis Infection and Upper Genital Tract Pathological Changes by Vaccine-Promoted Neutralizing Antibodies Directed to the VD4 of the Major Outer Membrane Protein.* Journal of Infectious Diseases, 2015. **212**(6): p. 978-989.

185. Jiang, P., et al., *Hepatitis B virus core antigen as a carrier for Chlamydia trachomatis MOMP multi-epitope peptide enhances protection against genital chlamydial infection.* Oncotarget, 2015. **6**(41): p. 43281-43292.

186. Waugh, C.A., et al., *Comparison of subcutaneous versus intranasal immunization of male koalas (Phascolarctos cinereus) for induction of mucosal and systemic immunity against Chlamydia pecorum.* Vaccine, 2015. **33**(7): p. 855-860.

187. Lorenzen, E., et al., *Intramuscular priming and intranasal boosting induce strong genital immunity through secretory IgA in minipigs infected with Chlamydia trachomatis.* Frontiers in Immunology, 2015. **6**(DEC).

188. Liu, S., et al., *Construction of recombinant HVT expressing PmpD, and immunological evaluation against chlamydia psittaci and Marek's Disease virus.* PLoS ONE, 2015. **10**(4).

189. Stary, G., et al., *A mucosal vaccine against Chlamydia trachomatis generates two waves of protective memory T cells.* Science, 2015. **348**(6241).

190. Badamchi-Zadeh, A., et al., *Intramuscular immunisation with chlamydial proteins induces chlamydia trachomatis specific ocular antibodies.* PLoS ONE, 2015. **10**(10).

191. Pal, S., O.V. Tatarenkova, and L.M. de la Maza, *A vaccine formulated with the major outer membrane protein can protect C3H/HeN, a highly susceptible strain of mice, from a Chlamydia muridarum genital challenge.* Immunology, 2015. **146**(3): p. 432-443.

192. Inic-Kanada, A., et al., *Delivery of a chlamydial adhesin N-PmpC subunit vaccine to the ocular mucosa using particulate carriers.* PLoS ONE, 2015. **10**(12).

193. Waugh, C., et al., *A prototype recombinant-protein based Chlamydia pecorum vaccine results in reduced chlamydial burden and less clinical disease in free-ranging koalas (Phascolarctos cinereus).* PLoS ONE, 2016. **11**(1).

194. Bøje, S., et al., *A multi-subunit Chlamydia vaccine inducing neutralizing antibodies and strong IFN-γ + CMI responses protects against a genital infection in minipigs.* Immunology and Cell Biology, 2016. **94**(2): p. 185-195.

195. Khan, S.A., et al., *Humoral immune responses in koalas (Phascolarctos cinereus) either naturally infected with Chlamydia pecorum or following administration of a recombinant chlamydial major outer membrane protein vaccine.* Vaccine, 2016. **34**(6): p. 775-782.

196. Badamchi-Zadeh, A., et al., *A multi-component prime-boost vaccination regimen with a consensus MOMP antigen enhances Chlamydia trachomatis clearance.* Frontiers in Immunology, 2016. **7**(APR).

197. Khan, S.A., et al., *Antibody and cytokine responses of koalas (Phascolarctos cinereus) vaccinated with Recombinant Chlamydial Major Outer Membrane Protein (MOMP) with two different adjuvants.* PLoS ONE, 2016. **11**(5).

198. Liang, M., et al., *Protective immunity induced by recombinant protein CPSIT_p8 of Chlamydia psittaci.* Applied Microbiology and Biotechnology, 2016. **100**(14): p. 6385-6393.

199. Bulir, D.C., et al., *Immunization with chlamydial type III secretion antigens reduces vaginal shedding and prevents fallopian tube pathology following live C. muridarum challenge.* Vaccine, 2016. **34**(34): p. 3979-3985.

200. Paes, W., et al., *Recombinant polymorphic membrane protein D in combination with a novel, second-generation lipid adjuvant protects against intra-vaginal Chlamydia trachomatis infection in mice.* Vaccine, 2016. **34**(35): p. 4123-4131.

201. Visan, L., et al., *Phosphate substitution in an AlOOH - TLR4 adjuvant system (SPA08) modulates the immunogenicity of Serovar E MOMP from Chlamydia trachomatis.* Human Vaccines and Immunotherapeutics, 2016. **12**(9): p. 2341-2350.

202. Inic-Kanada, A., et al., *A probiotic adjuvant lactobacillus rhamnosus enhances specific immune responses after ocular mucosal immunization with chlamydial polymorphic membrane protein C.* PLoS ONE, 2016. **11**(9).

203. Waugh, C., et al., *Treatment of Chlamydia-associated ocular disease via a recombinant protein based vaccine in the koala (Phascolarctos cinereus).* Biologicals, 2016. **44**(6): p. 588-590.

204. Hadad, R., et al., *Protection against genital tract Chlamydia trachomatis infection following intranasal immunization with a novel recombinant MOMP VS2/4 antigen.* APMIS, 2016. **124**(12): p. 1078-1086.

205. Wang, L., et al., *DNA plasmid vaccine carrying Chlamydia trachomatis (Ct) major outer membrane and human papillomavirus 16L2 proteins for anti-Ct infection.* Oncotarget, 2017. **8**(20): p. 33241-33251.

206. Koroleva, E.A., et al., *Chlamydial Type III Secretion System Needle Protein Induces Protective Immunity against Chlamydia muridarum Intravaginal Infection.* BioMed Research International, 2017. **2017**.

207. Ran, O., et al., *Recombinant protein CPSIT_0846 induces protective immunity against Chlamydia psittaci infection in BALB/c mice.* Pathogens and Disease, 2017. **75**(3).

208. Jiang, J., et al., *A protective vaccine against chlamydia genital infection using vault nanoparticles without an added adjuvant.* Vaccines, 2017. **5**(1).

209. Pal, S., et al., *The cationic liposomal adjuvants CAF01 and CAF09 formulated with the major outer membrane protein elicit robust protection in mice against a Chlamydia muridarum respiratory challenge.* Vaccine, 2017. **35**(13): p. 1705-1711.

210. Kuczkowska, K., et al., *Lactobacillus plantarum producing a Chlamydia trachomatis antigen induces a specific IgA response after mucosal booster immunization.* PLoS ONE, 2017. **12**(5).

211. Destrez, A., et al., *Effects of a chronic stress treatment on vaccinal response in lambs.* Animal, 2017. **11**(5): p. 872-880.

212. Wali, S., et al., *Chlamydial protease-like activity factor mediated protection against C. trachomatis in guinea pigs.* Immunology and Cell Biology, 2017. **95**(5): p. 454-460.

213. Pal, S., et al., *Comparison of the nine polymorphic membrane proteins of Chlamydia trachomatis for their ability to induce protective immune responses in mice against a C. muridarum challenge.* Vaccine, 2017. **35**(19): p. 2543-2549.

214. Müller, T., et al., *Vaccination with the polymorphic membrane protein A reduces Chlamydia muridarum induced genital tract pathology.* Vaccine, 2017. **35**(21): p. 2801-2810.

215. Wern, J.E., et al., *Simultaneous subcutaneous and intranasal administration of a CAF01-adjuvanted Chlamydia vaccine elicits elevated IgA and protective Th1/Th17 responses in the genital tract.* Frontiers in Immunology, 2017. **8**(MAY).

216. Jiang, P., et al., *Evaluation of tandem Chlamydia trachomatis MOMP multi-epitopes vaccine in BALB/c mice model.* Vaccine, 2017. **35**(23): p. 3096-3103.

217. Desclozeaux, M., et al., *Immunization of a wild koala population with a recombinant Chlamydia pecorum Major Outer Membrane Protein (MOMP) or Polymorphic Membrane Protein (PMP) based vaccine: New insights into immune response, protection and clearance.* PLoS ONE, 2017. **12**(6).

218. Pais, R., et al., *Rectal administration of a chlamydial subunit vaccine protects against genital infection and upper reproductive tract pathology in mice.* PLoS ONE, 2017. **12**(6).

219. Desclozeaux, M., et al., *Safety and immunogenicity of a prototype anti-Chlamydia pecorum recombinant protein vaccine in lambs and pregnant ewes.* Vaccine, 2017. **35**(27): p. 3461-3465.

220. O'Meara, C.P., et al., *Multistage vaccines containing outer membrane, type III secretion system and inclusion membrane proteins protects against a Chlamydia genital tract infection and pathology.* Vaccine, 2017. **35**(31): p. 3883-3888.

221. Ganda, I.S., et al., *Dendrimer-conjugated peptide vaccine enhances clearance of Chlamydia trachomatis genital infection.* International Journal of Pharmaceutics, 2017. **527**(1-2): p. 79-91.
